# Barriers to participation in mental health research: are there specific gender, ethnicity and age related barriers?

**DOI:** 10.1186/1471-244X-10-103

**Published:** 2010-12-02

**Authors:** Anna Woodall, Craig Morgan, Claire Sloan, Louise Howard

**Affiliations:** 1 Section of Women's Mental Health Health Services and Population Research Department, PO31 Institute of Psychiatry, King's College London, De Crespigny Park, SE5 8AF, London, UK; 2 Section of Social Psychiatry Health Service and Population Research Department, PO33 Institute of Psychiatry, King's College London, De Crespigny Park, SE5 8AF, London, UK

## Abstract

**Background:**

It is well established that the incidence, prevalence and presentation of mental disorders differ by gender, ethnicity and age, and there is evidence that there is also differential representation in mental health research by these characteristics. The aim of this paper is to a) review the current literature on the nature of barriers to participation in mental health research, with particular reference to gender, age and ethnicity; b) review the evidence on the effectiveness of strategies used to overcome these barriers.

**Method:**

Studies published up to December 2008 were identified using MEDLINE, PsycINFO and EMBASE using relevant mesh headings and keywords.

**Results:**

Forty-nine papers were identified. There was evidence of a wide range of barriers including transportation difficulties, distrust and suspicion of researchers, and the stigma attached to mental illness. Strategies to overcome these barriers included the use of bilingual staff, assistance with travel, avoiding the use of stigmatising language in marketing material and a focus on education about the disorder under investigation. There were very few evaluations of such strategies, but there was evidence that ethnically matching recruiters to potential participants did not improve recruitment rates. Educational strategies were helpful and increased recruitment.

**Conclusion:**

Mental health researchers should consider including caregivers in recruitment procedures where possible, provide clear descriptions of study aims and describe the representativeness of their sample when reporting study results. Studies that systematically investigate strategies to overcome barriers to recruitment are needed.

## Background

It is well established that the incidence, prevalence and presentation of mental disorders differ by gender, ethnicity and age. For example, men suffer from higher rates of alcohol dependency and antisocial personality disorder, and women have higher rates of depression, anxiety and somatic complaints [1,2]. Black and minority ethnic groups (BME) have a higher reported incidence of psychotic disorders [3], and are more likely to experience compulsory admission to psychiatric hospitals than whites [4] There is a higher incidence of schizophrenia in men compared with women [5] and men have poorer outcomes [6] but women are more likely to present with late onset psychotic disorders [7]. Alzheimer's Disease is more prevalent in women (reflecting the high proportions of women in the older adult population in industrialised countries)[8]. The validity of such findings is predicated on the assumption that recruitment of study participants is not overly influenced by sampling bias.

However, there is evidence that some groups are under-represented in mental health research [9] whereby insufficient numbers of participants are recruited to adequately represent a particular group of patients. For example, the National Survey of American Life: a study of racial, ethnic and cultural influences on mental disorders and mental health [10] found that initial refusal to participate was higher in African Caribbean participants, and the authors cite fears and suspicions concerning questions about possible immigration status as a reason for this. We also found that in a preliminary analysis of the Aetiology and Ethnicity of Schizophrenia and other Psychoses (AESOP) dataset [11], ethnic group and gender interacted to predict consent to participate; Black Caribbean men, and Black African women were more likely to refuse to take part in mental health research than their white British counterparts (Sloan & Morgan, personal communication).

There are a number of possible reasons why some groups are under-represented in mental health research. Firstly, until recently, investigators have tended to aim for homogeneity of study populations to avoid potential confounding. In addition women of childbearing age are also often routinely excluded from aetiological and intervention studies (e.g. neuro-imaging studies or drug trials) because of fears that if they are pregnant, or conceive during the study, the foetus will be put at risk [12], though there is evidence that this is changing [13,14]. Secondly, certain groups are less likely to access mental health services and will therefore not be available for studies that recruit through service contacts, e.g. BME patients and young men are less likely to access mental health services [15]. Also, gender and BME specific pathways to mental health care may also inhibit recruitment of these groups e.g. BME groups are more likely to have contact with mental health services via the criminal justice system [11]. The stigma associated with mental illness [16] may also affect willingness to participate in mental health research, both for participants who are mentally ill and healthy controls, particularly those from BME groups, or older potential participants[17]. Older adults may also be more physically frail, limiting their ability to attend research appointments and they are more likely to have chronic physical diseases which may mean they cannot be recruited into studies due to the exclusion criteria.

There is increased recognition of the importance of generalisability for study findings and the US National Institutes of Health (NIH) published amended guidelines in 2001 on the inclusion of women and minorities in research [18]. These guidelines state 'It is the policy of NIH that women and members of minority groups and their subpopulations must be included in all NIH-funded clinical research, unless a clear and compelling rationale and justification establishes to the satisfaction of the relevant Institute/Centre Director that inclusion is inappropriate with respect to the health of the subjects or the purpose of the research'. It is therefore vital that researchers know about potential barriers to participation in these groups and strategies that can effectively be used to overcome these barriers.

To our knowledge there has been no systematic review of barriers to participation in mental health research for different groups or how researchers have tried to overcome them. We therefore aimed to review the current literature on the nature of barriers to participation across different mental health studies with a focus on whether there are specific gender, age and ethnicity related barriers and to examine the evidence on the effectiveness of strategies used to overcome barriers.

## Methods

The research literature was searched using bibliographical electronic databases: Psyc-INFO, Medline and Embase, (1990-2008). The inclusion criteria were English language reports on barriers to recruitment in mental health research and strategies to address these on adult participants. The exclusion criteria were articles with a primary focus on eating disorders or substance abuse, non-empirical research articles and book chapters. "These databases were searched using search terms Mental disorders (Mapped term: exploded), and Recruitment$ and Research$ resulting in a retrieval of 661 articles across the three databases. Then, to narrow the search, the word barriers$, and finally, the term participation$ was added to the above search terms.

All of the articles retrieved (n = 157), using the above search terms, were subject to an initial screen. This involved reviewing the title and abstract of the retrieved article for subject relevance. After the search was conducted a primary researcher (CS) was responsible for selecting articles for review, and a second researcher (AW) repeated this process to check all relevant articles had been included. Where there was disagreement a senior author (LH) examined the paper and consensus was achieved after discussion. A circulatory approach to the review process was adopted [19], whereby the author moved between searching the literature base, analysing relevant studies to identify further studies and writing up; this is done so that the review remains firmly grounded in the available literature. Conceptual and methodological literatures are not readily subject to meta-analysis. It was anticipated that this review would include both qualitative and quantitative research therefore a more open and qualitative analysis of the results was considered appropriate [20].

**Figure 1:**
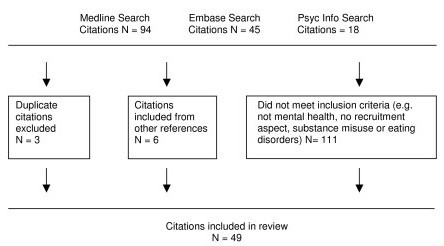
**Papers identified for review**.

We excluded articles relating to substance misuse only and eating disorders only (n = 11), articles unrelated to mental health research (n = 46), non-empirical research articles or book chapters (n = 20), and articles with no focus on recruitment (n = 34). A scan of the reference list, for potential additional articles of relevance was carried out for each paper and six additional papers were selected for initial inclusion in this review (see Figure 1).

## Results

**Figure 2:**
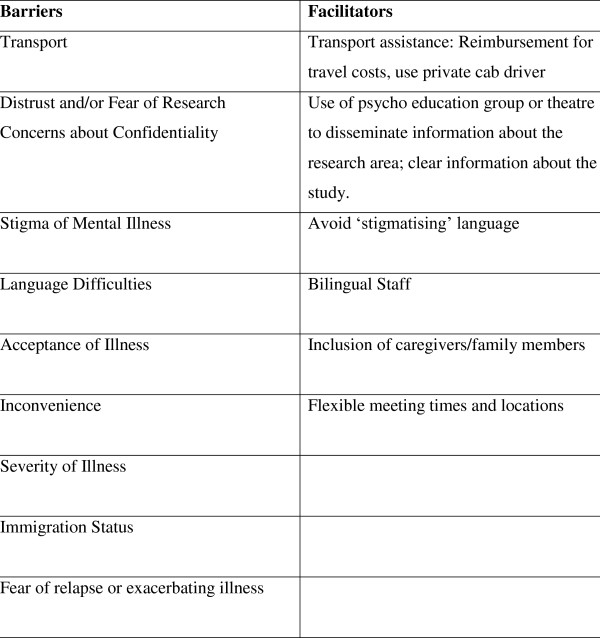
**Barriers and facilitators to recruitment**.

Forty-nine papers met the inclusion criteria of this review and were examined in detail (see additional file 1). The papers included in this review were diverse in type using a broad range of methodologies and participant populations. Papers are concerned with Dementia (n = 19), Schizophrenia (n = 7), depression (n = 15), Bipolar Disorder (n = 2), or mental illness in general (n = 8); one paper included patients with schizophrenia, depression, and bipolar disorder and therefore is included in each category. The methodologies used were qualitative (including surveys (n = 12), focus groups (n = 2), and semi-structured interviews (n = 8)), descriptions of different recruitment strategies in clinical trials (n = 7), and discussion of issues of recruitment and/or comparison of recruitment strategies within the same study (n = 26). One paper [21] compared the recruitment strategies of two RCTs on treatment models for depression. Additional files 2, 3, 4, 5, and 6 provide details of included studies for the different disorders, with information on the barriers identified in each study and the country from which papers originate. Figure 2 provides details of barriers and facilitators identified by the included studies.

### Identified Barriers

The barriers identified by researchers were broad ranging and included fear, suspicion and/or distrust of researchers [22-30], concerns about confidentiality [31], transportation difficulties [23,32-34], severity of illness[35-39], lack of financial reward [28,40], an increase in age - associated illness [35,37,41-45], inconvenience [23,33,46-48], fear of relapse as a result of participation [31,49], and the stigma of mental illness [24,26,26,26,27,44,50-53]. Also discussed were barriers that are not explicitly linked to the population being studied but rather the researchers themselves, which include competing academic centres studying the same group (which potentially increases participant refusal in one project due to participation in another), tensions between academic institutions and community centres, interdisciplinary differences [54], and relying on referrals from clinicians who have misconceptions about the research design and consequently have difficulty identifying and explaining the study to prospective patients [55].

In efforts to recruit minority ethnic groups specifically, a number of studies identified the stigma of mental illness [24,26,26,27,51,53] and distrust of researchers [10,22,56] as significant barriers. Results from a focus group with caregivers of patients with Alzheimer's disease indicated that the primary barrier for white participants was 'inconvenience' whereas for African American families it was more a general distrust of research [23]. Interviews with Chinese American Alzheimer caregivers also suggested that the social stigma associated with the disease was a barrier to research participation [51]. Language barriers [34] have also been cited as barriers to recruitment in minority ethnic groups. Immigration status proved to be a barrier to participation when attempting to recruit Mexican and Puerto Rican patients [26] and African American and African Caribbean migrant and second and older generation populations [10,26]. Loue and Sajatovic (2008) [26]discussed immigration status as also being a potential inhibiter to both initially contacting and remaining in contact with some patients as they were attempting to remain undetected by other authorities. In addition, the fear of being asked about immigration status within the context of a survey served as a challenge to initial recruitment [10].

Older participants are more difficult to recruit in some studies, and this can be due to a higher likelihood of physical illness[41]. Many of the studies done specifically with an older population also found that the difficulty of accepting a diagnosis of Dementia was a common barrier [22,23]. Through interviews it was found that caregivers of people with dementia were also concerned that the research activities would be harmful and cause excessive worry for the patient [51]. Rather than age itself serving as a barrier to recruitment, studies in relation to this factor were largely concerned with barriers to recruiting older adults into dementia research. It is interesting to note that one study of younger adults experiencing their first episode of mental illness also highlighted the difficulty of accepting a diagnosis as well as a decreased need for treatment as barriers [57].

In terms of gender, one study found that males were harder to recruit because they were less likely to endorse a diagnosis of depression because of the associated stigma [44]. However more men than women admitted to a psychiatric hospital recorded that they saw no reason to refuse participation when asked to consent to a range of hypothetical studies [28].

### Recruitment strategies to overcome barriers to participation

The recruitment strategies employed and discussed by researchers can be classified into three broad categories; suggestions for recruitment based on focus groups, interviews, and surveys with patients and others; author's opinions on what techniques they thought were helpful in their own recruitment; and strategies that were actually tested and measured in terms of effectiveness in increasing recruitment. It should be noted that strategies were not developed to recruit more effectively by age, gender or ethnicity specifically; instead more general strategies were described though some had a focus on minority recruitment.

Patient, caregiver, and professional suggestions on components essential for effective recruitment elicited from focus groups, interviews and surveys included: involving care givers [23]; emphasis on possible psycho-social benefits to participants including 'social and emotional support' from the research staff [23,31,58,59]; bilingual staff [51]; familiarity with researchers [31,60] and transportation assistance and incentives [28,31,32,61].

Some strategies are specifically relevant to certain contexts. For example, a study in the United States of America (USA) involved semi-structured interviews with patients who had and had not participated in mental health research to determine their main motivations and barriers to participation [31]. A strong motivating factor for patients with a diagnosis of schizophrenia was the offer of free medication; patients who were least happy with their current condition were more motivated to participate as they hoped that their symptoms would be alleviated.

Some researchers have provided suggestions on what recruitment techniques they believe were helpful in their research. These range from using bilingual staff (where studies have participants with other first languages) [26,26,34,62]; targeted marketing material [10,33,40,62,62,62]; assistance with travel costs and incentives [34,46,57,63,64]; flexible meeting times and locations [26,34,57], and avoiding the use of mental illness terms where possible to minimise the effect of the associated stigma [26,40,65].

Several studies describe different forms of outreach work designed to engage and consequently recruit ethnic minority participants. These range from hiring a specific 'outreach worker' (a person living within the community) [24] to assist researchers in meeting potential participants and advise study researchers on how to appropriately communicate, to (in certain contexts) ongoing consultation with community leaders [66]. Targeted marketing in the local communities was also employed [44,50]. While our review did not focus on retention of study participants, some authors commented on high retention rates which they attributed to certain strategies including the collection of alternative contacts for highly mobile subjects, birthday cards, and gifts for subjects [26,34]. One study used a group session to inform potential participants about a study [67]. The 45-minute presentation to potential participants described the nature of the RCT of supported employment. To be eligible for recruitment, potential participants had to attend four of these sessions; this was to ensure informed and committed people were recruited. The project met its recruitment target and had a good retention rate. However none of these strategies were formally evaluated.

A small number of studies have evaluated the efficacy of researcher/participant ethnic matching [10,21,68] and found little effect on recruitment rates. For example, Thompson et al (1996) found that ethnic matching of researchers to potential participants did not influence rates of refusal of interview completion for African American psychiatric inpatients. Recruitment data from a randomised trial to evaluate the effectiveness of a social model of care for patients with depression, anxiety or heavy drinking showed that ethnically matched recruiters were no more effective than experienced researchers with previous experience of conducting work with minorities and community member recruiters, with recruitment rates of 64%, 70%, and 77% respectively [21].

Other methods to increase minority recruitment that researchers have tried to evaluate included the distribution of educational pamphlets, a study newsletter and compensation for transportation costs in a study on dementia in black patients. When these strategies were introduced participation rates in a registry for Alzheimer's disease increased from 60 black patients (out of 607 potential participants) in year one to 150 in its second year [32]. Fritsch et al (2006) [69] increased minority recruitment rates (17% to 36%) over a 6 month period by exposing potential participants to a piece of educational theatre on Alzheimer's disease.

## Discussion

The main findings in terms of barriers identified ranged from practical factors (including transport, lack of financial reward, inconvenience, language difficulties, and immigration status), to more complex internal barriers (ranging from distrust of research and concerns about confidentiality, stigma of mental health, reluctance to accept a psychiatric diagnosis and fear of relapse). Strategies to overcome barriers to recruitment ranged from transport assistance and monetary incentives, bilingual staff, and inclusion of caregivers. Such practical solutions such as flexible meeting times, locations, and monetary incentives are relatively easy to implement in order to address barriers pertaining to transport, inconvenience, and financial difficulties. However other more complex barriers associated with the health, beliefs, and the culture of patients and/or carers were identified, which may be more difficult to overcome. We will therefore discuss these barriers in more detail.

### Fear and Concerns about Confidentiality

Through interviews with patients who had not consented to participate in a study of schizophrenia, Kaminsky et al (2003) [31] found that refusal was based on a fear of not knowing what was involved in the research, concerns about confidentiality of information and concerns that their personal information may be misused. Therefore clear, comprehensive explanations of the procedures involved in the study by researchers could serve to lessen any initial distrust. The recruitment targets were met in a study that utilised a research induction group to facilitate recruitment [67]. Within this forum there is the potential to provide sufficient information and respond to any concerns prospective participants have.

The mistrust and scepticism of mental health research found in ethnic communities [70] also inhibits prospective patients from volunteering to participate in research projects. This is in line with findings that ethnic minorities have a greater distrust of medical research in general [22,23,70]. Given that it is still discussed in the research and lay literature[71], this is perhaps a legacy from medical research projects such as the Tuskegee study, in which black men were not offered efficacious treatments for syphilis [72]. This scepticism of mental health research includes concerns about confidentiality of information shared [31] and perhaps the more deeply entrenched feelings of 'mental illness rooted in white oppression' [27].

The majority of studies were conducted in the USA and there did not appear to be significant differences in perceived barriers and facilitators to participation across countries. However we noted that three of the five studies based in England discussed the reluctance of clinicians to refer participants (due to a lack of confidence, skill, or misconception about research) as a significant barrier[73-75], in a way that was not explicitly discussed in studies conducted in the USA. This may indicate that research is more embedded in clinical practice there, or that less attention is paid to clinicians as potential barriers.

### Stigma of Mental Illness

The stigma associated with mental illness has been widely researched and seen as a factor effecting service engagement [16,76]. Stigma has also been cited as a factor affecting lower service use among ethnic minority groups [77-79]. The stigma attached to mental illness experienced by ethnic minorities such as African American [23] and Chinese American [51] communities were perceived to be a strong barrier to participation in mental health research. As ethnic minorities are less likely to access mental health services, reliance on clinical referrals and recruitment through services limits the potential pool of ethnic minority participants. Stigma and general distrust can also stem from a lack of understanding about the illness; some researchers who had difficulty recruiting Latino caregivers of persons with dementia cited a general lack of awareness and knowledge of dementia in the community as a significant barrier [62]. Other researchers deliberately avoided using terms associated with 'mental illness' and other 'stigmatizing diagnostic classifications' in their marketing and recruitment material, though ethically such techniques may be problematic if they are not transparent about the study aims.

A lack of knowledge about older age disorders has been highlighted as a potential barrier to participation in research into dementia [69]. Fritsch et al (2006) [69] significantly increased recruitment rate of African Americans into Alzheimer's disease research through exposure to live educational theatre on the topic. This type of community education can serve to lessen the stigma attached to a diagnosis of Alzheimer's disease.

### Acceptance of Diagnosis

Patients and family members' acceptance of a diagnosis is an important factor in gaining consent for participation. Studies of older adults with dementia or Alzheimer's Disease [22,23] found a reluctance to accept the diagnosis was a significant barrier. Not only did it prove to be a barrier at a patient level but clinicians themselves also resisted formally diagnosing Chinese American patients in an effort to reduce the perceived stigma of the diagnosis for that population [51]. Clinicians in this study reasoned that there was no need to give a potentially stigmatizing diagnosis if the elder was well cared for and not exhibiting any dangerous or violent behaviour. This consequently has implications for recruitment of these types of patients. A lack of insight or understanding, or an emotional need to deny a diagnosis, was found not only in elderly populations and their families but also with adolescents experiencing their first episode of psychosis or depression [57]. In one study males were more difficult to recruit due to their non-acceptance of their diagnosis of depression [44]. This is consistent with research that suggests males experience depression privately, unshared with others and attempt to alleviate it with little external help [80].

### Facilitators to Research Participation

Incentives to research participation such as the offer of free medication are relevant in countries where patients have difficulty in accessing and paying for health care. Even in countries with free healthcare people may see research as another avenue for help, as found by a United Kingdom (UK) based online survey on the views of patients regarding research participation (see http://www.healthtalkonline.org) [81]. This study also found that, of those people who were approached about mental health research, those who believed that their mental health may alter or be at risk of deteriorating as a result of participation were far less likely to participate in experimental studies and drug trials.

Results from studies that employed racial matching did not clearly indicate an improved consent rate from ethnic minority participants. Experience or 'cultural competence' appears to be more effective. However language specific marketing and use of researchers fluent in the minority groups' language is inevitably an effective component of recruitment [26,34,50,51,62].

Feedback from focus groups about research with older adults with dementia [23,50] highlight how important it is that family members and carers are actively involved in recruitment and research procedures, as the decision of consent will in large part be influenced by them. It is particularly important to include caregivers in the explanation and consent process as some caregivers have expressed worry that research participation could be potentially harmful to a patient [51].

A major motivating factor in participation in research appears to be the perception that the research may help others [31,50,61,82]. It follows that receiving research feedback from a study can reinforce this and show participants how they are contributing to the development of knowledge in that particular area [83]. Most studies agree that this is good practice and UK research ethics committees now routinely ask if this is planned. It is less clear how much time and resources should be allocated to providing detailed feedback. This is a longer-term strategy which aims to engage with the communities and educate them in what the research and their previous participation achieved; in the hope that this will lead to higher participation rates in future studies. This wider form of community awareness and engagement has been advocated by many authors [26,50,51,69] and is seen as crucial to recruitment. Participants also advocate this. In a study on schizophrenia, participants expressed a strong preference for being thoroughly debriefed about the purpose of each task at the conclusion of the study [61]. Outlining to a participant that they will receive some form of immediate feedback in the form of a debriefing may therefore increase the likelihood of consent.

### Implications

Research findings will not be generalisable if particular groups of patients are under-represented; research on the effectiveness of medication for certain ethnic groups for example has been limited [84] making it difficult to identify whether dosage should be altered or whether different drugs should be used. Study design ideally needs to reflect the population under investigation and where recruitment of large subgroups is not possible for practical reasons this should be addressed in the analysis e.g. by probability weighting in cost-effectiveness analyses.

Studies could also use more comprehensive datasets (e.g. administrative data) which could be helpful in triangulating research findings. Grant bodies could also try to ensure that planned research addresses the evaluation of recruitment strategies to ensure relevant groups are adequately represented.

### Limitations of Review

This literature search did not include hand-searching of relevant journals and formal rating of methodological quality was beyond the scope of this review - the diversity of the papers considered made this impractical. We found few studies that actually tested the effectiveness of a strategy making it difficult to attribute successful recruitment to a particular method. The lack of control groups in the majority of studies and comparisons of rates of recruitment could be affected by confounding factors. The literature to date illustrates how the majority of recruitment methods have not been formally evaluated and there is therefore a real gap in our understanding of barriers to participation. Much of the research in this area is at an exploratory stage only.

### Future Research Directions

Many researchers described barriers as discussion points or as a set of limitations after the results rather than something specifically considered in the study design. Where researchers did try to address potential barriers in their recruitment strategies, they did not formally evaluate these in any way. In view of the very limited evidence base on recruitment strategies to overcome barriers to participation in mental health research, we would recommend that future feasibility and pilot studies should include systematic evaluation of different recruitment strategies before starting a major study. Such development work is recommended by the UK Medical Research Council [85] and has been used successfully in other medical research settings [86]. We would also recommend that researchers clearly describe whether their sample is representative of the population of interest as recommended by the extended CONSORT guidelines[87].

Further research on age related barriers would be beneficial as little information was found on this. Other factors that may also be important barriers to participation such as level of education and socio-economic deprivation could also be explored in future research.

## Conclusions

There is little evidence on which recruitment strategies are effective for increasing rates of participation but studies did identify clear barriers which could be addressed by future researchers. For example, addressing difficulties with transportation is a clear and practical way to facilitate recruitment. Stigma, fear, and distrust were consistently found to be barriers across studies and consequently attempts to address these would also presumably increase recruitment. Transparency of the research project and a clear explanation of what is expected of participants may go a long way in dispelling fear and distrust. In addition, it is also important and worthwhile to be inclusive of caregivers and family members as in many cases they will be important contributors to decision making in participants' lives. This may also serve to ease some anxieties that prospective participants and their families may have about the impact of the research on the participants' health, and potential benefits. These could include allocation of different strategies in a randomised controlled trial would be beneficial. For example, a comparison of different marketing strategies and recruitment materials (e.g. information sheets) could be done whereby one set of materials uses less mental illness terminology that is potentially stigmatising, to determine if this increases recruitment rates. We would also recommend that researchers clearly describe whether their sample is representative of the population of interest. It is important for researchers to be aware and to try to recruit under-represented groups in future studies to ensure the validity of reported findings are not potentially undermined by sampling bias.

## Competing interests

The authors declare that they have no competing interests.

## Authors' contributions

CS and AW conducted the literature search and retrieved relevant articles. AW analysed and interpreted the content of the papers. LH and CM made substantial contributions to the interpretation of the data and revised the manuscript critically for important intellectual content. All authors helped draft the document, read and approved the final manuscript.

## Pre-publication history

The pre-publication history for this paper can be accessed here:

http://www.biomedcentral.com/1471-244X/10/103/prepub

## Supplementary Material

Additional file 1 **Appendix 1: Papers included in the systematic review**. A list of all references of the papers included in the review. Click here for file

Additional file 2 **Appendix 2 Table 1: Barriers to recruitment with regards to Schizophrenia**. A table summarising the information provided in the papers. Click here for file

Additional file 3 **Appendix 3: Table 2: Barriers to recruitment with regards to Bipolar Disorder**. A table summarising the information provided in the papers. Click here for file

Additional file 4 **Appendix 4: Table 3: Barriers to recruitment with regards to Depression**. A table summarising the information provided in the papers. Click here for file

Additional file 5 **Appendix 5: Table 4: Barriers to recruitment whereby the Mental Illness is not specified**. A table summarising the information provided in the papers. Click here for file

Additional file 6 **Appendix 6: Table 5: Barriers to recruitment with regards to Dementia**. A table summarising the information provided in the papers. Click here for file

## References

[B1] Anthony JC Warner LA Kessler RC Comparative Epidemiology of Dependence on Tobacco, Alcohol, Controlled Substances, and Inhalants: Basic Findings From the National Comorbidity Survey Experimental and Clinical Psycho pharmacology 1994 2 244 286 10.1037/1064-1297.2.3.244

[B2] Blazer DG Kessler RC McGonagle KA Swartz MS The prevalence and distribution of major depression in a national community sample: the National Comorbidity Survey American Journal of Psychiatry 1994 151 976 986 10.1176/ajp.151.7.979 8010383

[B3] Coid JW Kirkbride JB Barker D Cowden F Stamps R Yang M Raised incidence rates of all psychoses among migrant groups: Findings from the east london first episode psychosis study Archives of General Psychiatry 2008 65 1250 1258 10.1001/archpsyc.65.11.1250 18981336

[B4] Morgan C Mallett R Hutchinson G Bagalkote H Morgan K Fearon P Pathways to care and ethnicity. 1:Sample characteristics and compulsory admission British Journal of Psychiatry 2005 186 281 289 10.1192/bjp.186.4.281 15802683

[B5] McGrath J Saha S Chant D Welham J Schizophrenia: A Concise Overview of Incidence, Prevalence, and Mortality Epidemiologic Reviews 2008 30 67 76 10.1093/epirev/mxn001 18480098

[B6] Gur RE Petty RG Turetsky BI Gur RC Schizophrenia throughout life: sex differences in severity and profile of symptoms Schizophrenia Research 1996 21 1 12 10.1016/0920-9964(96)00023-0 8864248

[B7] Piccinelli M Gomez Homen F Gender differences in the epidemiology of affective disorders and schizophrenia 1997 Geneva: World Health Organization

[B8] Zhang M Katzman R Salmon D Jin H Cai G Wang Z The prevalence of dementia and Alzheimer's disease in Shanghai, China: Impact of age, gender, and education Annals of Neurology 2004 27 428 437 10.1002/ana.410270412 2353798

[B9] Iwamasa GY Sorocco KH Koonee DA Ethnicity and clinical psychology: A content analysis of the literature Clin Psychol Rev 2002 22 931 944 10.1016/S0272-7358(02)00147-2 12214331

[B10] Jackson JS Torres M Caldwell CH Neighbors HW Nesse RM Taylor RJ The National Survey of American Life: a study of racial, ethnic and cultural influences on mental disorders and mental health International Journal of Methods in Psychiatric Research 2004 13 196 207 10.1002/mpr.177 15719528 PMC6878295

[B11] Morgan C Dazzan P Jones P Harrison G Leff J Murray R First episode psychosis and ethnicity: initial findings from the AESOP study World Psychiatry 2006 5 40 46 16757995 PMC1472260

[B12] Howard L Webb R Abel K Antipsychotic drugs for pregnant and breastfeeding women with non-affective psychosis. Editorial Br Med J 2004 933 934 10.1136/bmj.329.7472.933 PMC524095 15499090

[B13] Goldkind SF Sahin L Gallauresi B Enrolling Pregnant Women in Research - Lessons from the N1N1 Influenza Pandemic The New England Journal of Medicine 2010 362 2241 2243 10.1056/NEJMp1003462 20554981

[B14] Macklin R The art of medicine Enrolling pregnant women in biomedical research The Lancet 2010 375 632 633 10.1016/S0140-6736(10)60257-7 20198725

[B15] Oliver MI Pearson N Coe N Gunnell D Help-seeking behaviour inmen and women with common mental health problems: cross-sectional study British Journal of Psychiatry 2005 186 297 301 10.1192/bjp.186.4.297 15802685

[B16] Thornicroft G Shunned: discrimination against people with mental illness 2006 Oxford: University Press: New York

[B17] Marwaha S Livingston G Stigma, racism or choice. Why do depressed ethnic elders avoid psychiatrists Journal of Affective Disorders 2002 72 257 265 10.1016/S0165-0327(01)00470-0 12450643

[B18] National Institutes of Health NIH Guidlines on the inclusion of women and minorities as subjects in clinical research 2001 NIH Guide 23 Ref Type: Report

[B19] Morrison B Lilford R How Can Action Research Apply to Health Services? Qualitative Health Research 2001 11 436 449 10.1177/104973201129119235 11521603

[B20] Morgan C Burns T Fitzpatrick R Pinfold V Priebe S Social exclusion andmental health Conceptual and methodological review British Journal of Psychiatry 2007 191 477 483 10.1192/bjp.bp.106.034942 18055950

[B21] Arean PA Alvidrez J Nery R Estes C Linkins K Recruitment and Retention of Older Minorities in Mental Health Services Research The Gerontologist 2003 43 36 44 12604744 10.1093/geront/43.1.36

[B22] Bachman DL Stuckey M Ebeling M Wagner MT Evans WJ Hirth V Establishment of a Predominantly African-American Cohort for the Study of Alzheimer's Disease Dement Geriatr Cogn Disord 2009 27 329 336 10.1159/000207446 19276625

[B23] Connell CM Shaw BA Holmes SB Foster NL Caregivers' attitudes toward their family members' participation in Alzheimer disease research: Implications for recruitment and retention Alzheimer Disease and Associated Disorders 2001 15 3 137 145 10.1097/00002093-200107000-00005 11522931

[B24] Gauthier MA Clarke WP Gaining and sustaining minority participation in longitudinal research projects Alzheimer Dis Assoc Disord 1999 13 S29 S33 10.1097/00002093-199904001-00008 10369515

[B25] Levkoff SE Levy BR Weitzman PF The matching model of recruitment. [References] Journal of Mental Health and Aging 2000 6 29 38

[B26] Loue S Sajatovic M Research with severely mentally ill Latinas: Successful recruitment and retention strategies J Immigr Minor Health 2008 10 145 153 10.1007/s10903-007-9063-9 17636454

[B27] Meinert JA Blehar MC Peindl KS Neal-Barnett A Wisner KL Bridging the gap: recruitment of African-American women into mental health research studies.[see comment] Academic Psychiatry 2003 27 21 28 10.1176/appi.ap.27.1.21 12824117

[B28] Zullino D Conus P Borgeat F Bonsack C Readiness to Participate in Psychiatric Research Can J Psychiatry 2003 48 480 484 12971019 10.1177/070674370304800709

[B29] Gallagher-Thompson D Rabinowitz Y Tang PCY Tse C Kwo E Hsu S Recruiting Chinese Americans for Dementia Caregiver Intervention Research: Suggestions for Success. [References] American Journal of Geriatric Psychiatry 2006 14 676 683 10.1097/01.JGP.0000221234.65585.f9 16861372

[B30] Patrick JH Pruchno RA Rose MS Recruiting research participants: a comparison of the costs and effectiveness of five recruitment strategies Gerontologist 1998 38 295 302 9640849 10.1093/geront/38.3.295

[B31] Kaminsky A Roberts LW Brody JL Influences upon willingness to participate in schizophrenia research: an analysis of narrative data from 63 people with schizophrenia Ethics Behav 2003 13 279 302 10.1207/S15327019EB1303_06 14680009

[B32] Ballard EL Nash F Raiford K Harrell LE Recruitment of Black elderly for clinical research studies of dementia: The CERAD experience Gerontologist 1993 33 561 565 8375688 10.1093/geront/33.4.561

[B33] Schlernitzauer M Bierhals AJ Geary MD Prigerson HG Stack JA Miller MD Recruitment methods for intervention research in bereavement-related depression. Five years' experience American Journal of Geriatric Psychiatry 1998 6 67 74 9469216

[B34] Miranda J Azocar F Organista KC Munoz RF Lieberman A Recruiting and retaining low-income Latinos in psychotherapy research Journal of Consulting & Clinical Psychology 1996 64 868 874 10.1037//0022-006x.64.5.868 8916613

[B35] Heun R Burkart M Maier W Selection biases during recruitment of patients and relatives for a family study in the elderly Journal of Psychiatric Research 1995 29 491 504 10.1016/0022-3956(95)00029-1 8642547

[B36] Launer LJ Wind AW Deeg DJH Nonresponse Pattern and Bias in a Community-based Cross-sectional study of cognitive functioning among the Elderly American Journal of Epidemiology 1994 139 803 812 8178793 10.1093/oxfordjournals.aje.a117077

[B37] Hofer A Hummer M Huber R Kurz M Walch T Fleischhacker WW Selection bias in clinical trials with antipsychotics Journal of Clinical Psychopharmacology 2000 20 6 699 702 10.1097/00004714-200012000-00019 11106145

[B38] Bowen J Hirsch S Recruitment rates and factors affecting recruitment for a clinical trial of a putative anti-psychotic agent in the treatment of acute schizophrenia Human Psychopharmacology 1992 7 5 337 341 10.1002/hup.470070507

[B39] Wittink MN Oslin D Knott KA Coyne JC Gallo JJ Zubritsky C Personal characteristics and depression-related attitudes of older adults and participation in stages of implementationof a multi-site effectiveness trial (PRISM-E) International Journal of Geriatric Psychiatry 2005 20 927 937 10.1002/gps.1386 16163743 PMC2771609

[B40] Warren PA Dunn L Jackson-Clark A The Medicare Alzheimer's Project in Portland, Oregon J Long Term Home Health Care 1991 10 20 27 10170803

[B41] Boersma F Eefsting JA van den Brink W van Tilburg W Characteristics of non-responders and the impact of non-response on prevalence estimates of dementia International Journal of Epidemiology 1997 26 1055 1062 10.1093/ije/26.5.1055 9363528

[B42] Cassidy EL Baird E Sheikh JI Recruitment and retention of elderly patients in clinical trials: Issues and strategies. [References] American Journal of Geriatric Psychiatry 2001 9 136 140 11316617

[B43] Christensen KJ Moye J Armson RR Kern TM Health Screening and Random Recruitment for Cognitive Aging Research Psychology and Aging 1992 7 204 208 10.1037/0882-7974.7.2.204 1610509 PMC4878448

[B44] Hinton L Zweifach M Oishi S Tang L Unutzer J Gender disparities in the treatment of late-life depression: qualitative and quantitative findings from the IMPACT trial American Journal of Geriatric Psychiatry 2006 14 884 892 10.1097/01.JGP.0000219282.32915.a4 17001028

[B45] Nazemi H Larkin AA Sullivan MD Katon W Methodological issues in the recruitment of primary care patients with depression International Journal of Psychiatry in Medicine 2001 31 277 288 10.2190/Q8BW-RAA7-F2H3-19BF 11841125

[B46] Cardemil EV Kim S Pinedo TM Miller IW Developing a culturally appropriate depression prevention program: the family coping skills program Cultural Diversity & Ethnic Minority Psychology 2005 11 99 112 10.1037/1099-9809.11.2.99 15884982

[B47] Scholle SH Peele PB Kelleher KJ Frank E Jansen-McWilliams L Kupfer D Effect of different recruitment sources on the composition of a bipolar disorder case registry Social Psychiatry and Psychiatric Epidemiology 2000 35 220 227 10.1007/s001270050231 10941997

[B48] Stack JA Paradis CF Reynolds CF III Houck PR Frank E Anderson B Does recruitment method make a difference? Effects on protocol retention and treatment outcome in elderly depressed patients Psychiatry Research 1995 56 17 24 10.1016/0165-1781(94)02617-R 7792338

[B49] Beebe LH What can we learn from pilot studies? Perspectives in Psychiatric Care 2007 43 213 218 10.1111/j.1744-6163.2007.00136.x 17894671

[B50] Aranda MP Racial and ethnic factors in dementia care-giving research in the US Aging and Mental Health 2001 5 Suppl 1 S116 S123 11513487 10.1080/13607860120044891

[B51] Hinton L Guo Z Hillygus J Levkoff S Working with culture: A qualitative analysis of barriers to the recruitment of Chinese-American family caregivers for dementia research J Cross Cult Gerontol 2000 15 119 137 10.1023/A:1006798316654 14618006

[B52] Levkoff SE Levy BR Weitzman PF The matching model of recruitment. [References] Journal of Mental Health and Aging 2000 6 29 38

[B53] Bonvicini KA The art of recruitment. The foundation of family and linkage studies of psychiatric illness Family Process 1998 37 153 165 10.1111/j.1545-5300.1998.00153.x 9693947

[B54] Levkoff SE Levy BR Weitzman PF The matching model of recruitment. [References] Journal of Mental Health and Aging 2000 6 29 38

[B55] Howard LM Leese M Byford S Killaspy H Cole L Lawlor C Methodological Challenges in Evaluating the Effectiveness of Women's Crisis Houses Compared With Psychiatric Wards: Findings From a Pilot Patient Preference RCT J Nerv Ment Dis 2009 197 722 727 10.1097/NMD.0b013e3181b97621 19829199

[B56] Morgan A Harris M Boyce P Wilhelm K Has social psychiatry met its Waterloo? Methodological and ethical issues in a community study Australian & New Zealand Journal of Psychiatry 1993 27 411 421 10.3109/00048679309075797 8250784

[B57] Furimsky I Cheung AH Dewa CS Zipursky RB Strategies to enhance patient recruitment and retention in research involving patients with a first episode of mental illness Contemporary Clinical Trials 2008 29 6 862 866 10.1016/j.cct.2008.07.005 18721902

[B58] Kaminsky A Roberts LW Brody JL Influences upon willingness to participate in schizophrenia research: an analysis of narrative data from 63 people with schizophrenia Ethics Behav 2003 13 279 302 10.1207/S15327019EB1303_06 14680009

[B59] Mastwyk M Ritchie CW LoGiudice D Sullivan KA Macfarlane S Carer impressions of participation in Alzheimer's disease clinical trials: What are their hopes? And is it worth it? International Psychogeriatrics 2002 14 1 39 45 10.1017/S1041610202008268 12094906

[B60] Norton MC Breitner JCS Welsh KA Wyse BW Characteristics of Nonresponders in a A Community Survey of the Elderly American Geriatric Society 1994 42 1252 1256 10.1111/j.1532-5415.1994.tb06506.x 7983287

[B61] Roberts LW Warner TD Anderson CT Smithpeter MV Rogers MK Schizophrenia research participants' responses to protocol safeguards: recruitment, consent, and debriefing Schizophr Res 2004 67 283 291 10.1016/S0920-9964(03)00101-4 14984889

[B62] Gallagher-Thompson D Singer LS Depp C Mausbach BT Cardenas V Coon DW Effective Recruitment Strategies for Latino and Caucasian Dementia Family Caregivers in Intervention Research American Journal of Geriatric Psychiatry 2004 12 484 490 15353386 10.1176/appi.ajgp.12.5.484

[B63] Skerritt U Pitt B Armstrong S O'Brien A Recruiting patients for drug trials: A difficult task Psychiatric Bulletin 1996 20 12 708 710 10.1192/pb.20.12.708

[B64] El-Khorazaty MN Johnson AA Kiely M El-Mohandes AA Subramanian S Laryea HA Recruitment and retention of low-income minority women in a behavioral intervention to reduce smoking, depression, and intimate partner violence during pregnancy BMC Public Health 2007 7 233 10.1186/1471-2458-7-233 17822526 PMC2020481

[B65] Peindl KS Wisner KL Successful recruitment strategies for women in postpartum mental health trials Journal of Psychiatric Research 2003 37 2 117 125 10.1016/S0022-3956(02)00086-9 12842165

[B66] Hendrie HC Lessons learned from international comparative crosscultural studies on dementia American Journal of Geriatric Psychiatry 2006 14 480 488 10.1097/01.JGP.0000192497.81296.fb 16731716

[B67] Drake RE Becker DR Anthony WA A research induction group for clients entering a mental health research project Hospital & Community Psychiatry 1994 45 487 489 10.1176/ps.45.5.487 8045547

[B68] Thompson EE Neighbors HW Munday C Jackson JS Recruitment and retention of African American patients for clinical research: an exploration of response rates in an urban psychiatric hospital Journal of Consulting & Clinical Psychology 1996 64 861 867 10.1037//0022-006x.64.5.861 8916612

[B69] Fritsch T Adams KB Redd D Sias T Herrup K Use of live theater to increase minority participation in Alzheimer disease research Alzheimer Disease and Associated Disorders 2006 20 2 105 111 10.1097/01.wad.0000213806.66811.ea 16772746

[B70] Corbie-Smith G Thomas SB George DM Distrust, Race, and Research Arch Intern Med 2002 162 2458 2463 10.1001/archinte.162.21.2458 12437405

[B71] The National Centre for Public Policy and Research EPA Sludge Tests a "Modern-Day Tuskegee Experiment" Children in Poor Black Neighborhoods Potentially Imperilled by EPA Studies 2008 Washington DC, The National Centre for Public Policy and Research Ref Type: Report

[B72] Jones J Bad Blood: The Tuskegee Syphilis Experiment 1981 New York: The Free Press

[B73] Daley AJ Winter H Grimmett C McGuinness M McManus R MacArthur C Feasibility of an exercise intervention for women with postnatal depression: A pilot randomised controlled trial British Journal of General Practice 2008 58 548 178 183 10.3399/bjgp08X277195 18399022 PMC2249793

[B74] Howard L de SI Tomlin Z Thornicroft G Donovan J Why is recruitment to trials difficult? An investigation into recruitment difficulties in an RCT of supported employment in patients with severe mental illness Contemporary Clinical Trials 2009 30 40 46 10.1016/j.cct.2008.07.007 18718555 PMC2626649

[B75] Mason VL Shaw A Wiles NJ Mulligan J Peters TJ Sharp D GPs' experiences of primary care mental health research: A qualitative study of the barriers to recruitment Family Practice 2007 24 5 518 525 10.1093/fampra/cmm047 17698979

[B76] Corrigan P How Stigma Interferes With Mental Health Care American Psychologist 2004 59 614 625 10.1037/0003-066X.59.7.614 15491256

[B77] Snowden L Barriers to Effective Mental Health Services Mental Health Services Research 2001 3 181 187 10.1023/A:1013172913880 11859964

[B78] Teng EJ Freidman LC Increasing mental health awareness and appropriate service use in older Chinese Americans: A pilot intervention Patient Education and Counseling 2009 76 143 146 10.1016/j.pec.2008.11.008 19124215

[B79] Mishra SI Lucksted A Gioia D Barnet B Baquet CR Needs and Preferences for Receiving Mental Health Information in an African American Focus Group Sample Community Mental Health 2009 45 117 126 10.1007/s10597-008-9157-4 PMC3618894 18633704

[B80] Warren LW Male Intolerance of Depression Clinical Psychology Reuiew 1983 3 156

[B81] healthtalkonline.org 2008 http://www.healthtalkonline.org/Ref Type: Electronic Citation

[B82] Roberts LW Warner TD Anderson CT Smithpeter MV Rogers MK Schizophrenia research participants' responses to protocol safeguards: recruitment, consent, and debriefing Schizophr Res 2004 67 283 291 10.1016/S0920-9964(03)00101-4 14984889

[B83] Aranda MP Racial and ethnic factors in dementia care-giving research in the US Aging and Mental Health 2001 5 Suppl 1 S116 S123 11513487 10.1080/13607860120044891

[B84] Fijal BA Kinon BJ Kapur S Stauffer VL Conley RR Jamal HH Candidate-gene association analysis of response to risperidone in African-American and white patients with Schizophrenia The Pharmacogenomics Journal 2009 9 311 318 10.1038/tpj.2009.24 19451915

[B85] Medical Research Council MRC Ethics Series Good Research Practice 2000 1 14 Ref Type: Report

[B86] Donavan J Mills N Smith M Brindle L Jacoby A Peters T Improving design and conduct of randomised trials by embedding them in qualitative research: ProtecT (prostate testing for cancer and treatment) study British Medical Journal 2002 325 766 770 10.1136/bmj.325.7367.766 12364308 PMC1124277

[B87] Schulz KF Altman DG Moher D CONSORT 2010 Statement: updated guidelines for reporting parallel group randomised trials British Medical Journal 2010 340 697 702 10.1136/bmj.c332 PMC3043330 21350618

