# Supplementary material for: Barriers to participation in mental health research: are there specific gender, ethnicity and age related barriers?
Source: BMC Psychiatry. 2010 Dec 2;10:103. doi: 10.1186/1471-244X-10-103 (PMC3016310; doi:10.1186/1471-244X-10-103)
Supplement: Additional file 1 — Appendix 1: Papers included in the systematic review. A list of all references of the papers included in the review. [file 1471-244X-10-103-S1.DOC]

**Appendix 1 Papers included in the systematic review**

Reference List

1. **Aranda, M. P.** **(2001)** Racial and ethnic factors in dementia care-giving research in the US. Aging and Mental Health.5(SUPPL.1)()(pp S116-S123), 2001.Date of Publication: 2001., S116-S123.

2. **Arean, P. A., Alvidrez, J., Nery, R.*, et al*** **(2003)** Recruitment and Retention of Older Minorities in Mental Health Services Research. The Gerontologist, **43**, 36-44.

3. **Bachman, D. L., Stuckey, M., Ebeling, M.*, et al*** **(2009)** Establishment of a Predominantly African-American Cohort for the Study of Alzheimer's Disease. Dement Geriatr Cogn Disord, **27**, 329-336.

4. **Ballard, E. L., Nash, F., Raiford, K.*, et al*** **(1993)** Recruitment of Black elderly for clinical research studies of dementia: The CERAD experience. Gerontologist, **33**, 561-565.

5. **Beebe, L. H.** **(2007)** What can we learn from pilot studies? Perspectives in Psychiatric Care, **43**, 213-218.

6. **Boersma, F., Eefsting, J. A., van den Brink, W.*, et al*** **(1997)** Characteristics of non-responders and the impact of non-response on prevalence estimates of dementia. International Journal of Epidemiology, **26**, 1055-1062.

7. **Bonvicini, K. A.** **(1998)** The art of recruitment. The foundation of family and linkage studies of psychiatric illness. Family Process, **37**, 153-165.

8. **Bowen, J. and Hirsch, S.** **(1992)** Recruitment rates and factors affecting recruitment for a clinical trial of a putative anti-psychotic agent in the treatment of acute schizophrenia. Human Psychopharmacology.7(5)()(pp 337-341), 1992.Date of Publication: 1992., 337-341.

9. **Cardemil, E. V., Kim, S., Pinedo, T. M.*, et al*** **(2005)** Developing a culturally appropriate depression prevention program: the family coping skills program. Cultural Diversity & Ethnic Minority Psychology, **11**, 99-112.

10. **Cassidy, E. L., Baird, E., and Sheikh, J. I.** **(2001)** Recruitment and retention of elderly patients in clinical trials: Issues and strategies. [References]. American Journal of Geriatric Psychiatry, **9**, 136-140.

11. **Christensen, K. J., Moye, J., Armson, R. R.*, et al*** **(1992)** Health Screening and Random Recruitment for Cognitive Aging Research. Psychology and Aging, **7**, 204-208.

12. **Connell, C. M., Shaw, B. A., Holmes, S. B.*, et al*** **(2001)** Caregivers' attitudes toward their family members' participation in Alzheimer disease research: Implications for recruitment and retention. Alzheimer Disease and Associated Disorders.15(3)()(pp 137-145), 2001.Date of Publication: 2001., 137-145.

13. **Daley, A. J., Winter, H., Grimmett, C.*, et al*** **(2008)** Feasibility of an exercise intervention for women with postnatal depression: A pilot randomised controlled trial. British Journal of General Practice.58(548)()(pp 178-183), 2008.Date of Publication: Mar 2008., 178-183.

14. **Drake, R. E., Becker, D. R., and Anthony, W. A.** **(1994)** A research induction group for clients entering a mental health research project. Hospital & Community Psychiatry, **45**, 487-489.

15. **El-Khorazaty, M. N., Johnson, A. A., Kiely, M.*, et al*** **(2007)** Recruitment and retention of low-income minority women in a behavioral intervention to reduce smoking, depression, and intimate partner violence during pregnancy. BMC Public Health, **7**, 233.

16. **Fritsch, T., Adams, K. B., Redd, D.*, et al*** **(2006)** Use of live theater to increase minority participation in Alzheimer disease research. Alzheimer Disease and Associated Disorders.20(2)()(pp 105-111), 2006.Date of Publication: Apr 2006., 105-111.

17. **Furimsky, I., Cheung, A. H., Dewa, C. S.*, et al*** **(2008)** Strategies to enhance patient recruitment and retention in research involving patients with a first episode of mental illness. Contemporary Clinical Trials.29(6)()(pp 862-866), 2008.Date of Publication: November 2008., 862-866.

18. **Gallagher-Thompson, D., Singer, L. S., Depp, C.*, et al*** **(2004)** Effective Recruitment Strategies for Latino and Caucasian Dementia Family Caregivers in Intervention Research. American Journal of Geriatric Psychiatry, **12**, 484-490.

19. **Gallagher-Thompson, D., Rabinowitz, Y., Tang, P. C. Y.*, et al*** **(2006)** Recruiting Chinese Americans for Dementia Caregiver Intervention Research: Suggestions for Success. [References]. American Journal of Geriatric Psychiatry, **14**, 676-683.

20. **Gauthier, M. A. and Clarke, W. P.** **(1999)** Gaining and sustaining minority participation in longitudinal research projects. Alzheimer Dis Assoc Disord, **13**, S29-S33.

21. **Hendrie, H. C.** **(2006)** Lessons learned from international comparative crosscultural studies on dementia. American Journal of Geriatric Psychiatry, **14**, 480-488.

22. **Heun, R., Burkart, M., and Maier, W.** **(1995)** Selection biases during recruitment of patients and relatives for a family study in the elderly. Journal of Psychiatric Research, **29**, 491-504.

23. **Hinton, L., Zweifach, M., Oishi, S.*, et al*** **(2006)** Gender disparities in the treatment of late-life depression: qualitative and quantitative findings from the IMPACT trial. American Journal of Geriatric Psychiatry, **14**, 884-892.

24. **Hinton, L., Guo, Z., Hillygus, J.*, et al*** **(2000)** Working with culture: A qualitative analysis of barriers to the recruitment of Chinese-American family caregivers for dementia research. J Cross Cult Gerontol, **15**, 119-137.

25. **Hofer, A., Hummer, M., Huber, R.*, et al*** **(2000)** Selection bias in clinical trials with antipsychotics. Journal of Clinical Psychopharmacology.20(6)()(pp 699-702), 2000.Date of Publication: 2000., 699-702.

26. **Howard, L., de, S., I, Tomlin, Z.*, et al*** **(2009)** Why is recruitment to trials difficult? An investigation into recruitment difficulties in an RCT of supported employment in patients with severe mental illness. Contemporary Clinical Trials, **30**, 40-46.

27. **Jackson, J. S., Torres, M., Caldwell, C. H.*, et al*** **(2004)** The National Survey of American Life: a study of racial, ethnic and cultural influences on mental disorders and mental health. International Journal of Methods in Psychiatric Research, **13**, 196-207.

28. **Kaminsky, A., Roberts, L. W., and Brody, J. L.** **(2003)** Influences upon willingness to participate in schizophrenia research: an analysis of narrative data from 63 people with schizophrenia. Ethics Behav, **13**, 279-302.

29. **Launer, L. J., Wind, A. W., and Deeg, D. J. H.** **(1994)** Nonresponse Pattern and Bias in a Community-based Cross-sectional study of cognitive functioning among the Elderly. American Journal of Epidemiology, **139**, 803-812.

30. **Levkoff, S. E., Levy, B. R., and Weitzman, P. F.** **(2000)** The matching model of recruitment. Journal of Mental Health and Aging, **6**, 29-38.

31. **Loue, S. and Sajatovic, M.** **(2008)** Research with severely mentally ill Latinas: Successful recruitment and retention strategies. J Immigr Minor Health, **10**, 145-153.

32. **Mason, V. L., Shaw, A., Wiles, N. J.*, et al*** **(2007)** GPs' experiences of primary care mental health research: A qualitative study of the barriers to recruitment. Family Practice.24(5)()(pp 518-525), 2007.Date of Publication: Oct 2007., 518-525.

33. **Mastwyk, M., Ritchie, C. W., LoGiudice, D.*, et al*** **(2002)** Carer impressions of participation in Alzheimer's disease clinical trials: What are their hopes? And is it worth it? International Psychogeriatrics.14(1)()(pp 39-45), 2002.Date of Publication: 2002., 39-45.

34. **Meinert, J. A., Blehar, M. C., Peindl, K. S.*, et al*** **(2003)** Bridging the gap: recruitment of African-American women into mental health research studies.[see comment]. Academic Psychiatry, **27**, 21-28.

35. **Miranda, J., Azocar, F., Organista, K. C.*, et al*** **(1996)** Recruiting and retaining low-income Latinos in psychotherapy research. Journal of Consulting & Clinical Psychology, **64**, 868-874.

36. **Morgan, A., Harris, M., Boyce, P.*, et al*** **(1993)** Has social psychiatry met its Waterloo? Methodological and ethical issues in a community study. Australian & New Zealand Journal of Psychiatry, **27**, 411-421.

37. **Nazemi, H., Larkin, A. A., Sullivan, M. D.*, et al*** **(2001)** Methodological issues in the recruitment of primary care patients with depression. International Journal of Psychiatry in Medicine, **31**, 277-288.

38. **Norton, M. C., Breitner, J. C. S., Welsh, K. A.*, et al*** **(1994)** Characteristics of Nonresponders in a A Community Survey of the Elderly. American Geriatric Society, **42**, 1252-1256.

39. **Patrick, J. H., Pruchno, R. A., and Rose, M. S.** **(1998)** Recruiting research participants: a comparison of the costs and effectiveness of five recruitment strategies. Gerontologist, **38**, 295-302.

40. **Peindl, K. S. and Wisner, K. L.** **(2003)** Successful recruitment strategies for women in postpartum mental health trials. Journal of Psychiatric Research.37(2)()(pp 117-125), 2003.Date of Publication: Mar 2003., 117-125.

41. **Roberts, L. W., Warner, T. D., Anderson, C. T.*, et al*** **(2004)** Schizophrenia research participants' responses to protocol safeguards: recruitment, consent, and debriefing. Schizophr Res, **67**, 283-291.

42. **Schlernitzauer, M., Bierhals, A. J., Geary, M. D.*, et al*** **(1998)** Recruitment methods for intervention research in bereavement-related depression. Five years' experience. American Journal of Geriatric Psychiatry, **6**, 67-74.

43. **Scholle, S. H., Peele, P. B., Kelleher, K. J.*, et al*** **(2000)** Effect of different recruitment sources on the composition of a bipolar disorder case registry. Social Psychiatry and Psychiatric Epidemiology, **35**, 220-227.

44. **Skerritt, U., Pitt, B., Armstrong, S.*, et al*** **(1996)** Recruiting patients for drug trials: A difficult task. Psychiatric Bulletin.20(12)()(pp 708-710), 1996.Date of Publication: 1996., 708-710.

45. **Stack, J. A., Paradis, C. F., Reynolds, C. F., III*, et al*** **(1995)** Does recruitment method make a difference? Effects on protocol retention and treatment outcome in elderly depressed patients. Psychiatry Research, **56**, 17-24.

46. **Thompson, E. E., Neighbors, H. W., Munday, C.*, et al*** **(1996)** Recruitment and retention of African American patients for clinical research: an exploration of response rates in an urban psychiatric hospital. Journal of Consulting & Clinical Psychology, **64**, 861-867.

47. **Warren, P. A., Dunn, L., and Jackson-Clark, A.** **(1991)** The Medicare Alzheimer's Project in Portland, Oregon. J Long Term Home Health Care, **10**, 20-27.

48. **Wittink, M. N., Oslin, D., Knott, K. A.*, et al*** **(2005)** Personal characteristics and depression-related attitudes of older adults and participation in stages of implementationof a multi-site effectiveness trial (PRISM-E). International Journal of Geriatric Psychiatry, **20**, 927-937.

49. **Zullino, D., Conus, P., Borgeat, F.*, et al*** **(2003)** Readiness to Participate in Psychiatric Research. Can J Psychiatry, **48**, 480-484.
