# Supplementary material for: Barriers to participation in mental health research: are there specific gender, ethnicity and age related barriers?
Source: BMC Psychiatry. 2010 Dec 2;10:103. doi: 10.1186/1471-244X-10-103 (PMC3016310; doi:10.1186/1471-244X-10-103)
Supplement: Additional file 2 — Appendix 2 Table 1: Barriers to recruitment with regards to Schizophrenia. A table summarising the information provided in the papers. [file 1471-244X-10-103-S2.PDF]

Appendix 2

Table 1: Barriers to Recruitment with regards to Schizophrenia

**Paper (authors)**	**Country of Origin/ Study population**	**Methods/ study design**	**Anticipated barriers**	**Barriers reported on recruitment**	**Strategies to over come these barriers/results [Proposed, Tested and, Used Strategies]**	**Methodological limitations.**	**Primary factor discussed/**
What can we learn from pilot studies? (Beebe, 2007) **[49]**	USA<br>Outpatient veterans with Schizophrenia (n = 10)<br>Age not listed.	Pilot study examining a 16-week walking program.	None discussed.	55% of eligible participants refused to participate citing the following reasons:<br>Lack of motivation.<br>Fear of pain.<br>Lack of knowledge about exercise benefits.<br>Given veteran benefits e.g. housing, the small financial remuneration offered may have not been a big enough incentive.	Possible strategies suggested by the authors: Provide alternatives to standard remuneration, allow additional recruitment time, use multiple recruitment sites, and combine research activities with existing veteran activities.	Pilot study with very small sample	General Barriers in veteran sample.
Recruitment rates and factors affecting recruitment for a clinical trial of putative anti psychotic agent in the treatment of acute schizophrenia (Bowen and Hirsch, 1992) **[38]**	ENGLAND<br>N = 44 Non-consenting patients screened to be in an anti psychotic drug trail.<br>Mean Age = 37.9 (consenters)<br>Mean Age = 37.6 (non consenters)	Clinical Trial<br>Asked non-consenters the reason refusal in the form of an open-ended question.	Severity of Illness	Minimum 3-week inpatient stay.<br>Experimental new medication.<br>Venepuncture procedure<br>And Electrocardiograms, due to delusional beliefs about blood, infection, and the cardiovascular system.<br>65.7% (44) of eligible patients did not consent to the study.	Emphasises the importance of putting resources to increase numbers in initial screening.	No analysis of recruitment strategies.	Patient reported barriers.
Selection bias in clinical trials with anti psychotics (Hofer *et al.* 2000) **[37]**	USA<br>Patients (18-65 years) consecutively admitted to inpatient units with a diagnosis of	Clinical trial<br>Recruits to the clinical trial (27) were compared with those who were ineligible or did not consent to the study (73).	Strict inclusion and exclusion criteria.	Recruited patients were younger and had been ill for a shorter period of time, and had fewer psychotic episodes.<br>44% did meet inclusion criteria.<br>29% refused to participate.	None discussed.	Patient's reasons for non-consent were not explored.	Comparison of consent and non-consent patients.

Appendix 2
Table 1: Barriers to Recruitment with regards to Schizophrenia

**Paper (authors)**	**Country of Origin/ Study population**	**Methods/ study design**	**Anticipated barriers**	**Barriers reported on recruitment**	**Strategies to over come these barriers/results [Proposed, Tested and, Used Strategies]**	**Methodological limitations.**	**Primary factor discussed/**
	schizophreniform or schizophrenia. N=200						
Influences upon willingness to participate in Schizophrenia Research: An Analysis of Narrative Data From 63 People with Schizophrenia (Kaminsky *et al.* 2003) **[58]**	USA<br>Patients (White, Black, Hispanic, and Native American) with a diagnosis of schizophrenia.<br>Involved in research. N=28<br>Not involved in research N = 35<br>Mean Age = 43	Structured interview about research participation decisions and willingness.	Lack of trust of research.<br>Lack of clarity about the use of personal information and confidentiality protections.	Fewer non-whites were in involved in research group compared with the not involved in research group ($\chi^2 = 6.61$, $p > 0.05$).<br>The main barriers identified by the participants' were:<br>• Concerns about the confidentiality.<br>• The fear of unknown research expectations.<br>• Fear of potential relapse due to participation.<br>• Receive placebo and potential of worsening symptoms.<br>• Burden of participation (tasks are difficult, no compensation and or/ transport)	Based on participant's comments researchers need to should address the following to improve recruitment: participation:<br>• Psychosocial benefits including increasing self-awareness, interaction with others and helping other's with mental health problems.<br>• Access to potentially better medication.<br>• Monetary incentives, transportation and health care assistance.<br>• Knowing clearly what the research involves made deciding to partake easier.<br>• Familiarity with doctors and researchers.	Despite having an ethnically diverse no ethnic specific barriers to participation were explored.	General barriers perceived by patients.
Research with severely mentally ill Latinas: Successful recruitment and	USA<br>Female Mexican and Puerto Rican (18-50 years) with a diagnosis of	Study of context of HIV risk behaviour.<br>Participants had a baseline interview and follow-up each year for	Trust.<br>Immigration status.<br>Language differences.	Non- legal immigration status.<br>Community level distrust of research<br>Participant isolation	Strategies used:<br>Multi lingual staff.<br>Small incentives.<br>Avoid the term 'mental illness' in study marketing material.	The effectiveness of a strategy is not assessed.	Gender: Female<br>Ethnicity

Appendix 2
Table 1: Barriers to Recruitment with regards to Schizophrenia

**Paper (authors)**	**Country of Origin/ Study population**	**Methods/ study design**	**Anticipated barriers**	**Barriers reported on recruitment**	**Strategies to over come these barriers/results [Proposed, Tested and, Used Strategies]**	**Methodological limitations.**	**Primary factor discussed/**
retention strategies (Loue and Sajatovic, 2008) **[26]**	schizophrenia, bipolar disorder, or major depression.	two years in addition to 100 hours of observation (researcher shadowing them).		Stigma<br>Competing obligations<br>Severity of psychopathology	Emphasis on retention strategies: Alternative contacts obtained with consent to contact them to assist in tracking them down participants.<br>Birthday and holiday cards sent to participants		
Recruiting research participants: A comparison of costs and effectiveness strategies. (Patrick *et al.* 1998) **[30]**	USA<br>Caregivers of patients with Schizophrenia, mental retardation or other developmental difficulties.<br>N=841<br>Mean Age = 65.2	Mixed methods<br>Main study: Qualitative interview study.<br>Quantitative: Examination of participant characteristics recruited through five different strategies:<br>Formal service agencies,<br>Support groups,<br>Snowball referrals,<br>Media approaches, and<br>Modified demographic sampling unit.	Fear and mistrust of research.	Few barriers were reported as the study ran to time and met the recruitment target.<br>African American women were more costly to recruit in terms of staff time and monetary recourses. i.e. the average cost of recruiting an African American woman was $ 54.15 compared to $29.92 .	African American's and Caucasians were recruited into the study via different methods ($F(4, 836) = 55.65$, $p <.0001$)<br>African American's were less likely to be recruited through snow ball (nomination of potential participants by study participants) sampling and more likely to be recruited though modified demographic sampling (letters and follow up phone calls to a sample in a community with a high proportion of African Americans).<br>Snowball sampling referrals were significantly older than media referred participants.	Could have included some analysis of participant views on their recruitment strategy i.e. why this worked for them rather than just describing the characteristics of participants recruited in different ways.	Ethnicity
Schizophrenia research participants responses to protocol safeguards: Recruitment, consent, and debriefing	MEXICO<br>Participants in a research protocol with a diagnosis of Schizophrenia.<br>N=28<br>Mean Age = 40.75	Survey & Semi structured interview study on attitudes to biomedical research including responses to vignettes describing research studies.	None discussed	Burden of participation, either in terms of fear or logistical difficulty.	Future strategies could focus on what participant's identified as their main incentives; the expectation of benefit from the study (21%, n = 6), incentives (14%, n = 4), and sense of altruism (11%, n = 3).<br>The majority of people decided to participate in the study on first	Those that refused to participate were not included and may have been able to provide more information on barriers to recruitment.<br>No details are	General barriers perceived by patients

Appendix 2
Table 1: Barriers to Recruitment with regards to Schizophrenia

**Paper (authors)**	**Country of Origin/ Study population**	**Methods/ study design**	**Anticipated barriers**	**Barriers reported on recruitment**	**Strategies to over come these barriers/results [Proposed, Tested and, Used Strategies]**	**Methodological limitations.**	**Primary factor discussed/**
(Roberts *et al.* 2004) **[61]**					hearing about it (54%, n = 14) or after talking with the researcher (31%, n = 8), rather than after reading the consent form (8%, n = 2). Therefore instilling trust in an initial referral or invitation is an important recruitment strategy.	given about the nature of the research the patients participated in.	
