# Supplementary material for: Barriers to participation in mental health research: are there specific gender, ethnicity and age related barriers?
Source: BMC Psychiatry. 2010 Dec 2;10:103. doi: 10.1186/1471-244X-10-103 (PMC3016310; doi:10.1186/1471-244X-10-103)
Supplement: Additional file 3 — Appendix 3: Table 2: Barriers to recruitment with regards to Bipolar Disorder. A table summarising the information provided in the papers. [file 1471-244X-10-103-S3.PDF]

Appendix 3
Table 2: Barriers to Recruitment with regards to Bipolar Disorder.

**Paper (authors)**	**Country of Origin/ Study population**	**Methods/ study design**	**Anticipated barriers**	**Barriers reported on recruitment**	**Strategies to over come these barriers/results [Proposed, Tested and, Used Strategies]]**	**Methodological limitations.**	**Primary factor discussed/**
Research with severally mentally ill Latinas: Successful recruitment and retention strategies (Loue and Sajatovic, 2008)See Schizophrenia Table. **[26]**							
Effect of different recruitment sources on composition of a bi polar case registry. (Scholle *et al.* 2000) **[47]**	USA<br>Members of a National voluntary bipolar case registry (April 1995- October 1997) sponsored by a university psychiatry department in Pittsburgh USA. 88% of registrants were in psychiatric treatment.<br>Locally recruited participants were 92% Caucasian, 61% Female.<br>National registrants were 63 % female, & 94 % Caucasian.	Quantitative analysis of demographics of registry patients.<br>Recruitment methods: Health professionals; patient support groups; general public relations and other i.e. (word of mouth).<br>Mean ages are listed in the paper across recruitment source.	Recruitment source.	Local Registrants:<br>Not significant however patients recruited through patient support groups tended to be older, better educated, more likely to be married, and Caucasian.<br>National Registrants:<br>Those recruited through the internet were more likely to be younger and better educated.	Local registrants (n = 684) were primarily recruited through health professionals (44%), and a general public relations effort by the centre staff (22%). Recruitment by public relations efforts was the most expensive method of recruitment.<br><br>National registrants (n = 823) were mostly recruited through the internet (32%) and patient support groups (31%).<br><br>No sig gender differences were cited by recruitment source.		Barriers to registering on the voluntary bipolar case registry.
