# Supplementary material for: Barriers to participation in mental health research: are there specific gender, ethnicity and age related barriers?
Source: BMC Psychiatry. 2010 Dec 2;10:103. doi: 10.1186/1471-244X-10-103 (PMC3016310; doi:10.1186/1471-244X-10-103)
Supplement: Additional file 4 — Appendix 4: Table 3: Barriers to recruitment with regards to Depression. A table summarising the information provided in the papers. [file 1471-244X-10-103-S4.PDF]

Appendix 4

Table 3: Barriers to Recruitment with regards to Depression.

**Paper (authors)**	**Country of Origin/ Study population**	**Methods/ study design**	**Anticipated barriers**	**Barriers reported on recruitment**	**Strategies to over come these barriers/results [Proposed, Tested and, Used Strategies]**	**Methodological limitations.**	**Primary factor discussed/**
Recruitment and Retention of Older Minorities in Mental Health Services Research. (Arean *et al.* 2003) **[21]**	USA<br>**Study 1** PEPUP (Psychotherapy effectiveness project for underserved primary care Patients):<br>N= 71<br>Mean Age = 65<br>Randomised trial of 3 types of psychosocial interventions for depression.<br>**Study 2** PASS: (Patients access to social services):<br>Randomised trial of a social service model of care. Participants meet the criteria for depression, anxiety or heavy drinking.<br>N=121<br>Mean Age = 75	Comparison of recruitment rates across the two studies.<br>**Strategy 1:** PEPUP Study: Traditional scientific methods of recruitment: Provider Referral and Self-referral in response to newspapers, TV, and radio programs.<br>**Strategy 2:** PASS Study: Consumer centred approach to recruitment**:** Personalised mailing, face to face recruitment within the neighbourhood primary care clinic, provider referral, and ethnically matched staff.	Respondent burden<br>Stigma<br>Alienation of the research team from the community.	Not specifically reported.	No significant difference was found (χ^2 = .82, ns) between traditional and consumer centred methods with regards to recruiting older minorities. [The consumer centred approach resulted in greater retention of older minorities over time ($\chi^2 = 6.20$, $p > 0.05$)].<br>In Study 2, minorities (88%) were more likely to participate through face-to-face recruitment then whites (42%).<br>Being an experienced or community recruiter resulted in greater agreement to participate than simply ethnically matching the recruiter ($\chi^2 = 37.00$, $p < .001$).	Recruitment rates were compared across two different studies and therefore could be due to other factors e.g. differences in patient populations, case identification method. Also the 'consumer centred approach' had similar techniques as the 'traditional approach' eg provider referral therefore the two approaches are not entirely dissimilar making valid comparisons difficult.	Age<br>Ethnicity
Developing a culturally appropriate depression prevention	USA<br>Low income Latino mothers in a depression prevention	Intervention study:<br>6 CBT based group sessions, and 2 family sessions.<br>Recruitment was done	Lower access to mental health services may make this group harder to recruit	Of 103 people expressed interest in the project, 47 attended an initial assessment. Of these 33 consented to the project,	Programme offered:<br>• Flexibility with regards to language choice of sessions.<br>• Culturally relevant		Ethnicity

Appendix 4

Table 3: Barriers to Recruitment with regards to Depression.

**Paper (authors)**	**Country of Origin/ Study population**	**Methods/ study design**	**Anticipated barriers**	**Barriers reported on recruitment**	**Strategies to over come these barriers/results [Proposed, Tested and, Used Strategies]**	**Methodological limitations.**	**Primary factor discussed/**
programme: the family coping skills programme. (Cardemil *et al.* 2005) **[46]**	programme.<br>N=33<br>Age not listed.	through waiting rooms at a free paediatric clinic, a local community health centre, local community organisations, word of mouth		those 14 people cited scheduling difficulties as the reason. for non-consent.	content.<br>• Transportation costs and onsite child care.<br>• Program provided 'culturally competent' staff as defined as 'having experience with, and being comfortable with interacting with low-income Latina women.		
Recruitment and retention of elderly subjects in clinical trials. (Cassidy *et al.* 2001) **[42]**	USA<br>Patients with a diagnosis of Depression (> 65)<br>N=19	Clinical Trail to determine the tolerability of the medication Zoloft and whether it effectively treated symptoms of major depressive disorder in older patients.	Travel arrangements<br>Strict study exclusion criteria	Prevalence of the disorder being studied.<br>Lack of available funding<br>Age<br>Complexity of the study<br>Lack of sufficient compensation.	Early strategies resulted in no recruits.<br>• Newspaper adds, mailing to local primary care physicians and senior centres, university out patient clinic. Adverts on pharmacy bags<br>Later recruitment efforts turned to radio ads, which resulted in recruiting 19 subjects.	Small sample size.	Age
Feasibility of an exercise intervention for women with postnatal depression: a pilot randomised controlled trial. (Daley *et al.* 2008) **[73]**	UNITED KINGDOM<br>Women experiencing depression >16 years with youngest child was < 12 months old.<br>N = 38	Participants recruited through 4 different routes; identified by GP's, identified by 'mother and baby unit', identified by health visitors, and self referral. Cases randomised to exercise intervention group or usual care.	Limited time due to demands of new baby.	Poor response from GP's in assisting with identification of cases.	Recruitment from the mother and baby unit proved most successful; 42.9% (12) of those identified agreed to participate as opposed to the GP response rate 28.1% (27).	Small sample	Gender
Recruitment and retention of low	USA<br>Pregnant African	Intervention Study: Behavioural and	Distrust of research.	Denial of need for help.<br>Lack of interest.	Recruitment training (rapport building).	Differences in participants	Ethnicity

Appendix 4

Table 3: Barriers to Recruitment with regards to Depression.

**Paper (authors)**	**Country of Origin/ Study population**	**Methods/ study design**	**Anticipated barriers**	**Barriers reported on recruitment**	**Strategies to over come these barriers/results [Proposed, Tested and, Used Strategies]**	**Methodological limitations.**	**Primary factor discussed/**
income minority women in a behavioural intervention to reduce smoking, depression, and intimate partner violence during pregnancy. (El-Khorazaty *et al.* 2007) **[64]**	American and Latino Women > 18 years and of 28 weeks gestational age or less.<br>N = 1191	Counselling Intervention Trial.	Transportation.	Claims to be beyond 28 weeks gestation.<br>Not enough time.	Cooperation from clinic staff.<br>Financial incentives	retained and lost to follow up were looked at rather than those who were unable to be recruited initially.	
Strategies to enhance patient recruitment and retention in research involving patients with a first episode of mental illness. (Furimsky *et al.* 2008) **[57]**	CANADA<br>Study 1: RCT in First Episode Psychosis. Comparison of Mobile Treatment Team and Outpatient Clinic.<br>Study 2: Clinical Trial for adolescent depression conducted in 5 tertiary care centres examining the benefit of citalopram.<br>Age not listed.	Review of recruitment challenges in two clinical trials (first episode depression or first episode psychosis)	None discussed.	Patient and family acceptance of diagnosis.<br>Burden of study visits (missing school cited as big reason).<br>Reluctance of clinician referral.<br>Decreased need for treatment.	Introduce idea of research at first contact with mental health services.<br>Streamlining clinical and research assessments.<br>Monetary incentives and transportation costs.<br>Flexible appointment times.	Does not discuss ethnic or gender factors that may influence participation.<br>Effectiveness of strategies is not evaluated.	Age
Gender Disparities in the Treatment of Late –Life depression:	USA<br>Informants (physicians, depression care	Effectiveness trial:<br>Mixed methods:<br>Quantitative: Referral rates	None discussed	Quantitative Results:<br>• Men were significantly less likely to be referred to the study via their	None discussed.	No data are reported on those who refused to enter the study.	Age<br>Gender

Appendix 4

Table 3: Barriers to Recruitment with regards to Depression.

**Paper (authors)**	**Country of Origin/ Study population**	**Methods/ study design**	**Anticipated barriers**	**Barriers reported on recruitment**	**Strategies to over come these barriers/results [Proposed, Tested and, Used Strategies]**	**Methodological limitations.**	**Primary factor discussed/**
Qualitative and Quantitative findings form the IMPACT Trial. (Hinton *et al.* 2006) **[44]**	managers, recruiters) on the IMPACT (a multi-site trial of a disease management program for late-life depression in primary care).<br>N= 30<br>Age not listed.	Qualitative: Interviews with referring physicians, depression care managers, and study recruiters.		primary care physician ($\chi^2[1] = 10.88$, p<0.001).<br>• Men were significantly less likely than women to endorse depression symptoms which makes them more difficult to refer to the study ($\chi^2[1] = 21.24$, p<0.001).<br>Qualitative Results:<br>• Practitioner's state that engaging men in a discussion about depression is more difficult than with women making referrals to treatment and research difficult.<br>• Older men in particular are less likely to accept they have depression because of traditional masculine values and the stigma associated with mental illness.		Qualitative data reflects the perspectives of clinicians and recruiters rather than patients' themselves.	
Research with severally mentally ill Latinas: Successful recruitment and retention strategies (Loue and							

Appendix 4

Table 3: Barriers to Recruitment with regards to Depression.

**Paper (authors)**	**Country of Origin/ Study population**	**Methods/ study design**	**Anticipated barriers**	**Barriers reported on recruitment**	**Strategies to over come these barriers/results [Proposed, Tested and, Used Strategies]**	**Methodological limitations.**	**Primary factor discussed/**
Sajatovic, 2008)<br>**[26]**							
GP's experiences of primary care mental health research: a qualitative study of the barriers to recruitment.<br>(Mason et al., 2007).<br>**[75]**	UK<br>GP' from five primary care trusts in South West England who were collaborating with the University of Bristol on an RCT recruiting patients with depression.<br>N=41<br>Mean Age = 46.4	Qualitative Study: Semi-structured interviews to investigate perceived barriers among GPs towards referring patients presenting with depression to a RCT.	None discussed	Three major themes identified:<br>1. Concern about protecting the patient and the doctor-patient relationship.<br>2. Perceived lack of skill and confidence of GP's.<br>3. Priority given to clinical and administrative tasks over research participation.	None discussed.	GP's from practices that had not agreed to collaborate with the RCT were not included and therefore more sceptical views of research my not be represented.	Barriers as perceived by GP's.
Recruiting and retaining Low income Latinos in Psychotherapy research.<br>(Miranda *et al.* 1996)<br>**[34]**	USA<br>Low income Latin American Women across 4 studies.<br>1:<br>Women with high levels of depressive symptoms, histories of abuse with anxiously attached infants.<br>N-100<br>2:<br>No participant characteristics provided.	Two prevention intervention studies and two treatment studies.<br>1.<br>Anxiously attached infants were randomly assigned to intervention (infant-parent psychotherapy) or to a control group.<br>2.<br>Depression prevention project: Randomised control trial with primary care patients.<br>3.	Availability and access to health care services.	Lack of insurance, childcare, and transportation.<br>Language Difficulties<br>Dominant male led families, where the male needs to be consulted for the female to participate in research.<br>Latinos found the traditional therapy environment 'cold'.	Similar strategies for recruitment were employed across the studies:<br>Strong ties with the medical clinics referring the patients to the study (the principal investigator was also a faculty member at the Department of Medicine at San Francisco General Hospital).<br>Transportation provided.<br>Home Visits<br>Childcare costs.<br>Bi lingual and bi cultural staff.<br>Cash reimbursement.<br>Retention Strategies: Mothers and Babies received birthday cards.<br>Recruiter remembered specific	The researchers attribute successful recruitment to these strategies especially bilingual and bicultural research which while it is likely, these method s were not specifically evaluated for their effectiveness.	Ethnicity

Appendix 4

Table 3: Barriers to Recruitment with regards to Depression.

**Paper (authors)**	**Country of Origin/ Study population**	**Methods/ study design**	**Anticipated barriers**	**Barriers reported on recruitment**	**Strategies to over come these barriers/results [Proposed, Tested and, Used Strategies]**	**Methodological limitations.**	**Primary factor discussed/**
	3: Women with depression with young children. N = 7 4: Primary care patients with a diagnosis of depression.	Group Cognitive-Behaviour Therapy combined with Infant-Parent Psychotherapy. 4. Randomised trial of group cognitive-behavioural therapy or group cognitive-behavioural therapy supplemented with case management			details about family to discuss with participants.		
Methodological issues in the recruitment of primary care patients with depression. (Nazemi *et al.* 2001) **[45]**	USA Patients from 3 residency-based primary care clinics approached to partake in a treatment-outcome study of minor depression and dysthymia. N=609 Age not listed	Comparison of two recruitment strategies: Waiting Room (WR) screening and screening after physician referral (PR).	None discussed.	Those who refused screening in the WR group (26% n = 837) were older (Mean = 50.43 ± 17.50 vs. Mean = 47.18 ± 17.85; t(3097) = 4.40, p < .01) and more often male (28.6% vs. 24.5%, Two=tailed Fisher's Exact Test, p<.05).	Given that male's seemed to more likely be referred via physician's the study suggests close working relationships with practitioners.	Fairly homogeneous sample of predominantly white and female participants.	Gender
Successful recruitment strategies for women in postpartum mental health trials. (Peindl and Wisner, 2003) **[65]**	USA Women who were pregnant or in the postpartum period. N= 589 Age not listed.	Descriptive study examining recruitment rates of different strategies (professional referral, mass mailings, and media advertisements) for recruiting women into one of two clinical trials	None discussed.	Recruitment depended largely on professional referrals.	Develop relationships in the community and establish a referral network of obstetricians emphasising an interest in their patients 'wellbeing' rather than 'mental illness'. Professional referral was the best source of participants (49% (77) in treatment study and 44% (57) in		Gender

Appendix 4
Table 3: Barriers to Recruitment with regards to Depression.

**Paper (authors)**	**Country of Origin/ Study population**	**Methods/ study design**	**Anticipated barriers**	**Barriers reported on recruitment**	**Strategies to over come these barriers/results [Proposed, Tested and, Used Strategies]**	**Methodological limitations.**	**Primary factor discussed/**
		for the prevention and treatment of postpartum major depression.			prevention study).		
Recruitment methods for intervention research in bereavement-related depression. Five years experience. (Schlernitzauer *et al.* 1998) **[33]**	USA Patients (≥ 50) meeting diagnostic criteria for non-delusional unipolar major depression or intermittent minor depression experienced 6 months before to 2 years after the death of a loved one N= 65 Age not listed.	Comparison of strategies to recruit elderly subjects with bereavement related depression in an ongoing randomized, placebo-controlled clinical trial testing the efficacy of nortriptyline and interpersonal psychotherapy over 5 years.	Low socio economic status	Of the 441 patients were screened 329 met the inclusion criteria but didn't enter the study due to the following: Contacted the study 'out of curiosity' but denied help. Not willing to change current treatment of depression to that of the trials. Unwilling to travel to appointments. Medical burden (medication). Time constraints.	Advertisements in local newspapers (35) and letters of information to people identified as having lost someone from the Obituaries in The Pittsburgh Post-Gazette (9) yielded the largest amount of recruits. Information letters sent to health providers did not account for any recruits, researcher suspect this is in part due to clinician's bias against the use of antidepressant medication for bereavement related depression.		Age
Does recruitment method make a difference, Effects on protocol retention and treatment outcome in the elderly depressed patients (Stack *et al.* 1995) **[48]**	USA Elderly patients (M age = 67.5) in a clinical trial of maintenance therapies in late life depression. N=125	Comparison of participants who self referred via media campaign (56) or were clinically referred (69).	None discussed.	Clinically referred group. Higher proportion were African American, had a lower level of education, fewer economic resources, and higher chronic medical burden.	Despite demographic differences, there was no difference between treatment outcome, time to response, rate of attrition or treatment refusal between clinically referred and self referred patients.	Findings are not applicable to other groups	Age Ethnicity SES
Personal Characteristics and depression-related attitudes of	USA All patients aged ≥ 65 who had an appointment with	Information on personal characteristics and attitudes regarding depression and its	Patients who didn't agree with a statement that depression should	Patient's who rated their health as 'fair' or 'poor' ($p=0.01$; adjusted p-value $=0.03$), their social support as	As the notion that depression is best treated by medication only emerged as a predictor of participation at the stage when	The patients were not specifically asked why they did or did not	Age

Appendix 4
Table 3: Barriers to Recruitment with regards to Depression.

**Paper (authors)**	**Country of Origin/ Study population**	**Methods/ study design**	**Anticipated barriers**	**Barriers reported on recruitment**	**Strategies to over come these barriers/results [Proposed, Tested and, Used Strategies]**	**Methodological limitations.**	**Primary factor discussed/**
older adults and participation in stages of implementation of a multi-site effectiveness trial (PRISM-E). (Wittink *et al.* 2005) **[39]**	one of 34 primary care clinicians. A random subset of patients was selected from each practice each week. N = 8423	treatment were obtained from all potential participants at each step of the Primary Care Research in Substance Abuse and Mental Health for the Elderly treatment effectiveness trial.	be treated with medication would be less likely to participate.	‘some’, ‘little’ or ‘not at all’ (p=0.03; adjusted p-value=0.09) were less likely to complete the baseline diagnostic interview. The mean BOMC (Brief Orientation Memory Concentration) test of patients who didn’t participate was significantly lower (M = 4.4; p= 0.01); adjusted p-value 0.03) than those who did (5.5). Those who went on to meet the mental health specialist reported less social support (p=0.01; adjusted p-value=0.03) and endorsed the statement that depressions should be treated with medication.	treatment was being offered and not at the earlier stages of recruitment this study supports the idea that extensive costs on assessing and addressing attitudes about treatment are not necessary early on in the recruitment process but are best utilized closer to the randomisation process.	participate in various stages of recruitment.	
