# Supplementary material for: Barriers to participation in mental health research: are there specific gender, ethnicity and age related barriers?
Source: BMC Psychiatry. 2010 Dec 2;10:103. doi: 10.1186/1471-244X-10-103 (PMC3016310; doi:10.1186/1471-244X-10-103)
Supplement: Additional file 5 — Appendix 5: Table 4: Barriers to recruitment whereby the Mental Illness is not specified. A table summarising the information provided in the papers. [file 1471-244X-10-103-S5.PDF]

Appendix 5
Table 4: Barriers to Recruitment whereby the Mental Illness is not specified.

**Paper (authors)**	**Country of Origin/ Study population**	**Methods/ study design**	**Anticipated barriers**	**Barriers reported on recruitment**	**Strategies to over come these barriers/results [Proposed, Tested and, Used Strategies]**	**Methodological limitations.**	**Primary factor discussed/**
The art of recruitment: the foundation of family and linkage studies of psychiatric illness (Bonvicini, 1998) **[53]**	USA<br>Participants for family linkage studies.<br>Case study was Italian American family.	Discussion of recruitment issues for family linkage studies, and a case study.	Family dynamics may present a barrier for participation	Families worried about being labelled mentally ill.	Identification of important family members, which may differ across cultures.<br>Elderly grandmother recruited first as matriarch of the family. This process facilitated successful recruitment of other members of the family.	Case study findings are not generalisable.	Family dynamics
A research induction group for clients entering a mental health research project. (Drake *et al.* 1994) **[67]**	USA<br>Participants entering a supported employment intervention (age 18-65), with severe and persistent mental illness, out of hospital for at least 1 month,<br>N= 143<br>Age not listed.	Description of the use of a research induction group in an experimental study of two models of supported employment for person with severe mental illness.	Many clinical trials fail because of the inability to recruit subjects or because the early attrition of subjects reduces the design to a quasi-experimental design.	88 people who had attended the group and decided not to partake in the study seemed to have made informed decisions with reasons ranging from deciding they were not ready for a competitive job and not liking on of the proposed interventions.	Psycho-educational discussion group (research induction group) met weekly for 45 minutes throughout an 18-month recruitment period at two participating mental health centres. The group allowed prospective participants to learn about the project in terms of the interventions offered, the research interviews, consent and confidentiality. Patients had to attend 4 of these sessions before enrolling in the study.<br>5 participants dropped out within a year of starting the program indicating a good retention rate.	Ethnicity of sample is not discussed.	Induction Group
Why is recruitment to trials difficult? An investigation into recruitment difficulties in an	ENGLAND<br>Trial and clinical staff involved in a RCT of supported employment for patients with	Qualitative study during the recruitment phase of an RCT of patients with severe mental illness. Interviews were conducted with trial and	None discussed	Clinician's misconceptions about RCT's, lack of equipoise and misunderstanding of the trial arm when describing the study to prospective patients.	Clinician and patient involvement in study designs and proposed recruitment strategies would be helpful and serve to address clinician's potential ambivalence and lack of experience with	Small sample of interviews.	Barrier's as perceived by clinicians.

Appendix 5
Table 4: Barriers to Recruitment whereby the Mental Illness is not specified.

**Paper (authors)**	**Country of Origin/ Study population**	**Methods/ study design**	**Anticipated barriers**	**Barriers reported on recruitment**	**Strategies to over come these barriers/results [Proposed, Tested and, Used Strategies]**	**Methodological limitations.**	**Primary factor discussed/**
RCT of supported employment in patients with sever mental illness (Howard *et al.* 2009) **[74]**	severe mental illness.<br>N= 4 trial staff	clinical staff as well as two workshops with clinical staff on the recruitment process and perceived difficulties with the trial.		Clinician's applied their own eligibility criteria on patients when making referrals.	research.		
The National survey of American life: A study of racial ethnic and cultural influences on mental disorders and mental health (Jackson *et al.* 2004) **[10]**	USA<br>Adult Sample<br>African-Americans n= 3570<br>Afro- Caribbean's, n= 1623<br>Non Hispanic whites n= 1,006<br>Adolescent sample (13-17 years): Afro-Caribbean and African American n=1,200	Survey	None discussed	Afro-Caribbean sample had higher refusal rates 'Especially after September 11th'.<br>Cited reason: Fears and suspicions concerning questions about possible immigration status.	Press releases issued to local black newspapers and radio stations in the cities with the highest refusal rates. Investigators also spoke on local talk shows to increase participation.<br>• 300 African American interviewers were hired and trained to implement race matching.<br>• Additional study staff had to be hired two years into the study to assist the in screening and recruitment due to the unanticipated personal problems such as deaths of close family members and other personal problems, which occur disproportionately more among at-risk ethnic groups, such as African-Americans.	.	Ethnicity
Bridging the Gap:	USA	Evaluation/discussion of	Distrust of the	African Americans did not	From the information gathered at	Research findings	Gender

Appendix 5
Table 4: Barriers to Recruitment whereby the Mental Illness is not specified.

**Paper (authors)**	**Country of Origin/ Study population**	**Methods/ study design**	**Anticipated barriers**	**Barriers reported on recruitment**	**Strategies to over come these barriers/results [Proposed, Tested and, Used Strategies]**	**Methodological limitations.**	**Primary factor discussed/**
Recruitment of African American Women into Mental Health Research studies. (Meinert *et al.* 2003) **[27]**	African American women residing in (ranging in age from late teens to late 70s) Cleveland USA.<br>Aims:<br>To provide education about mental health to African American women, and to increase minority recruitment for a NIMHR funded study of anti depressant use during pregnancy.	the experience of making contact with African American women's support group, and subsequent conference designed for AA women to elicit their views and attitudes towards mental health research.	medical system, stigma of mental illness, economic disadvantages, communication and cultural differences, a general lack of awareness of study programs.	access mental health because of inadequate health insurance, and burden of childcare responsibilities.<br>Personal issues such as mental illness kept in the family, or keeping a stiff upper lip,<br>Black mental illness associated with white oppression.<br>Suspicion of mental health services.<br>Preference to seek help from prayer and spirituality, and talking with friends.	the conference the authors suggest the following types of recruitment strategies:<br>• Develop contacts of significant individuals or organizations representative of your target population.<br>• Interact personally with members of the minority group to address any misunderstandings about the project.<br>• Listen to individuals concerns about the research topic.<br>• Identify 'non-traditional' supports as sources of recruitment. Eg African American spiritual institutions.	are not empirically tested. I.e. we do not know how many women were recruited for the subsequent antidepressant and pregnancy study.<br>Methods used to elicit information from the conferences were not systematic.	Ethnicity
Has Social Psychiatry met its waterloo: Methodological issues in a community study (Morgan *et al.* 1993) **[56]**	AUSTRALIA<br>Socially Disadvantaged females recruited from a 'door knock' approach of government housing in Sydney.<br>N = 193	Longitudinal Community- Based Study investigating the influence of psychosocial factors in the development of minor psychiatric disorder among women.<br>Information collected	Participant has to let researcher into their home, lengthy interview, invasion of privacy, and concerns over confidentiality.	2918 Dwellings allocated to the door knock. A member of the household was spoken to in 2247. 433 Women were identified as eligible. 193 consented.<br>People were suspicious of researchers and thought they represented a government	Provide evidence that the study was being run by a legitimate institution.<br>Assurances of confidentiality.<br>Attention to language e.g. uses the terms 'stress' and 'coping' rather than mental illness.	No information on ethnicity was collected.	General Barriers.

Appendix 5
Table 4: Barriers to Recruitment whereby the Mental Illness is not specified.

**Paper (authors)**	**Country of Origin/ Study population**	**Methods/ study design**	**Anticipated barriers**	**Barriers reported on recruitment**	**Strategies to over come these barriers/results [Proposed, Tested and, Used Strategies]**	**Methodological limitations.**	**Primary factor discussed/**
	Mean Age = 30.4	from participants 5 times over 2 years.		agency particularly the Department of Housing and that the researchers will provide personal information to the Department.			
Recruitment and Retention of African Patients for Clinical Research: An exploration of response Rates in an Urban Psychiatric hospital. (Thompson *et al.* 1996) **[68]**	USA<br>Psychiatric residents served as interviewers over the course of the study.<br>N=15<br>White and African American psychiatric inpatients.<br>62% male<br>78% AA<br>N= 960<br>Age not listed.	The impact on patient recruitment by racially matching interviewers (psychiatric residents) and patients for a study on the influence of ethnicity was tested.	Distrust of researchers as an effect of historical research as a part of institutional racism. Researchers are viewed as taking but not giving anything to the community.	Differing interviewer/treating physician ethnicity may result in decreased participation rates.	Cultural sensitivity examined by matching interviewers with potential participants on the basis of skin colour or ethnic group membership.<br>Results:<br>• No differences found between whites and African American for refusal or completion to participate<br>• Ethnic matching did not increase interview completion or refusal rates for African American patients.<br>• Diagnosis had the most impact, patients with a diagnosis of Schizophrenia patients (n = 162; 19.6%%) were statistically less likely to refuse to participate than those with mood disorders (n = 53; 10.8%).		Ethnicity
Readiness to	SWITZERLAND	Survey	None discussed.	Patients with F2 diagnosis	Participants reported the following	The study	Gender and

Appendix 5
Table 4: Barriers to Recruitment whereby the Mental Illness is not specified.

**Paper (authors)**	**Country of Origin/ Study population**	**Methods/ study design**	**Anticipated barriers**	**Barriers reported on recruitment**	**Strategies to over come these barriers/results [Proposed, Tested and, Used Strategies]**	**Methodological limitations.**	**Primary factor discussed/**
participate in Psychiatric Research. (Zullino *et al.* 2003) **[28]**	Consecutive patients admitted to a psychiatric university hospital interviewed about their (hypothetical) willingness to participate in different types of studies.<br>N = 100<br>Mean Age = 36.3			were generally more reluctant to participate in studies. They more often refused post marketing drug trials (35.7% vs 13.0%; $P<0.05$), double-blind trials (42.9% vs 18.8%; $P < 0.05$), blood sampling (21.4% vs 7.2%), and repeated interviews (21.4% vs 4.4%). They were less convinced that they would benefit from a newer treatment (50% vs 71%; $P < 0.05$) and indicated less often "to help science and other patients" as their reason for participation (67.9% vs 94.2%; $P < 0.01$). Likewise, they relied less frequently on their family physician to discuss participation (50% vs 71%; $P < 0.05$)More men mentioned lack of financial compensations as a reason for non-participation (12.8% vs 3.4%; $p< .05$).	themes to inform future recruitment strategies:<br>• Participants relied mainly on their treating physician, family physician (n = 63, 64.9%), or hospital physician (n = 52, 53.6%) when contemplating participation. This indicates that inclusive working with practitioners would be beneficial.<br>• The most common reason to participate was to help science progress and help future patients	described a hypothetical situation in which patients were asked to participate in a trail. In real situations the patients may respond differently.	age
