# Supplementary material for: Barriers to participation in mental health research: are there specific gender, ethnicity and age related barriers?
Source: BMC Psychiatry. 2010 Dec 2;10:103. doi: 10.1186/1471-244X-10-103 (PMC3016310; doi:10.1186/1471-244X-10-103)
Supplement: Additional file 6 — Appendix 6: Table 5: Barriers to recruitment with regards to Dementia. A table summarising the information provided in the papers. [file 1471-244X-10-103-S6.PDF]

Appendix 6
Table 5: Barriers to Recruitment with regards to Dementia.

**Paper (authors)**	**Country of Origin/ Study population**	**Methods/ study design**	**Anticipated barriers**	**Barriers reported on recruitment**	**Strategies to over come these barriers/results [Proposed, Tested and, Used Strategies]**	**Methodological limitations.**	**Primary factor discussed/**
Racial and ethnic factors in dementia care-giving research in the US (Aranda, 2001) **[50]**	USA<br>Dementia caregivers of older racial and ethnic minorities, particularly of Latino origin.<br>Description of three studies with varied methodologies; demonstration project, focus group, and cross-sectional survey.	Demonstration Project:<br>El Portal Latino Alzheimer's Project.<br>Aim of the project was to enhance the Latino community's capacity to provide culturally and linguistically competent educational, medical, social, and supportive services for Latino dementia- affected persons and caregivers.<br><br>Bilingual Focus Groups:<br>Latino consumers of primary medical care. > 65.<br>Ethnicity: Mexican or Mexican American<br>N = 35.	Individual:<br>Denial or underestimation of ones ability to effect change as a result of participation in research<br>Socio- cultural<br>Differences in disease-treatment-rehabilitation paradigms due to linguistic, cultural, socio-historical, and minority group status factors.<br>Economic<br>Financial and transactional costs associated with participation in all junctures of the research process.<br>Scientific:<br>Scientific processes that result in the exclusion or under-research of certain sub populations that compromise social, justice/redistributive	Demonstration Project<br>• Stigma of mental illness.<br>Focus Groups<br>• Discuss participation with other family members before finalising consent to participate. This may prolong initial phase of obtaining informed consent as it may span over several decision makers.	Demonstration project:<br>• Spanish and English help lines, bilingual staff, and electronic media advertising. Spanish marketing presentations to consumer provider groups, community fairs, and consumer to consumer referrals.<br>• A Spanish company did project marketing, brochures advertising the project were handed out by an ex-caregiver (Spanish) and advisory councils of community volunteers were established to provide advice to the project, and increase awareness of the project through work of mouth.<br>• These efforts resulted in a 50% increase in first time inquirers for AD issues from Latinos over a 45 month period.<br>Focus group:<br>Future strategies would need to address the following themes:	There is no evidence on which particular strategy was the most effective.	Ethnicity

Appendix 6
Table 5: Barriers to Recruitment with regards to Dementia.

**Paper (authors)**	**Country of Origin/ Study population**	**Methods/ study design**	**Anticipated barriers**	**Barriers reported on recruitment**	**Strategies to over come these barriers/results [Proposed, Tested and, Used Strategies]**	**Methodological limitations.**	**Primary factor discussed/**
			goals.<br><br><u>Administrative</u>: Administrative concerns that promote overly cautious attitudes and practices leading to delays in the inclusion of diverse populations.		• Feed back the research results to the community<br>• Value of "el compromise" or giving back was a major motivator for participation. Participants said they participated in an effort to improve services for others.<br>• Participating in research is in part dependent upon the agreement of others in the participant's life i.e. family members.<br>• If clinical staff are to refer study participants they must have detailed knowledge about the study as unsuitable groups may be referred and suitable participants missed.		
Establishment of a predominantly African-American cohort for the study of Alzheimer's disease. (Bachman *et al.* 2009)	USA<br>Patients with Alzheimer's Disease seeking medical care in clinical venues in South Carolina.<br>N= 122<br>Mean Age = 73	Epidemiology<br>Longitudinal study of risk factors for Alzheimer's Disease.	None discussed.	The difference between African American patients who were approached and volunteered (43.4%) compared to whites (70.3%) was significantly different (p=0.0025) despite all patients having a pre-existing relationship with the	Utilising geographically diverse sites where minorities already receive medical care and are more likely to have established trusting clinician- patient relationships.	The patients themselves were not asked about their reasons for non-participation.	Ethnicity

Appendix 6
Table 5: Barriers to Recruitment with regards to Dementia.

**Paper (authors)**	**Country of Origin/ Study population**	**Methods/ study design**	**Anticipated barriers**	**Barriers reported on recruitment**	**Strategies to over come these barriers/results [Proposed, Tested and, Used Strategies]**	**Methodological limitations.**	**Primary factor discussed/**
**[22]**				recruiters at each site.<br>General scepticism about research and reluctance to discuss Alzheimer's Disease was cited by the researcher's as reason for low African American participation.			
Recruitment of black elderly for clinical research studies of dementia: The CERAD Experience (Ballard *et al.* 1993)<br>**[32]**	USA<br>Clinical personnel at nine sites of the Consortium to Establish a Case Registry for Alzheimer's Disease (CREAD) (cooperating network of 27 US medical centres with associated sites abroad).	Semi Structured Survey of 9 sites to identify barriers to recruiting black subjects.	None discussed.	Lack of information about the disease.<br>Transportation difficulties<br>Perceived in-accessibility of the clinic site.<br>Resistant to external help.	In response to survey findings the following was implemented:<br>• Educational brochures to increase community awareness of Alzheimer's disease in '6th grade' English and Spanish.<br>• Reimbursement for transportation costs offered.<br>This resulted in an increase in black subjects from 60 cases and 12 controls to 150 cases and 28 controls over two years	While there was an increase in recruitment rates, it is unclear how much of this is a direct result of the implemented strategies and not just in line with previous recruitment rates.	Ethnicity
Characteristics of non-responders and the impact of non-response on prevalence estimates of dementia. (Boersma *et al.* 1997)<br>**[41]**	NETHERLANDS<br>Patients from eight general practices in a rural area near Zwolle.<br>Mean Age 74<br>Refusers N = 369<br>Responders N = 2191	Responders and people who refused to participate in a cross-sectional community study were compared with regards to in socio-demographic characteristics and cognition.	None discussed.	Older subjects were more likely to refuse.<br>Subjects living at home not an institution were more likely to refuse.	None discussed	Discusses non-responders more in terms of how they would affect rates in prevalence studies rather than suggesting or testing any strategies to improve recruitment.	Age

Appendix 6
Table 5: Barriers to Recruitment with regards to Dementia.

**Paper (authors)**	**Country of Origin/ Study population**	**Methods/ study design**	**Anticipated barriers**	**Barriers reported on recruitment**	**Strategies to over come these barriers/results [Proposed, Tested and, Used Strategies]**	**Methodological limitations.**	**Primary factor discussed/**
Health screening and random recruitment for cognitive aging research. (Christensen *et al.* 1992) **[43]**	USA<br>Adult's > 60<br>N= 315	Survey<br>Recruited by random digit dialling to be screened for participation in a neuropsychological research project.	Increase in age and a decrease in health status may increase participation.	People who agreed to participate were, younger, better educated and more likely to be males [R^2 of .01, F (3,199) = 11.16p < 0.0005].	None discussed.	Ethnicity data not obtained.<br>Overview of the sample they recruited rather than explicitly looking at barriers and strategies.	Gender and age
Care giver attitudes towards their family members participation in Alzheimer's disease research: Implications for recruitment and retention (Connell *et al.* 2001) **[23]**	USA<br>50 Care givers of Alzheimer's patients.<br>Ethnicity:<br>African Americans (AA) N =12<br>White American (WA) N = 38<br>Mean Age: 64	Focus group	Distrust of research.<br>Lack of perceived efficacy of the research	General Barriers:<br>Transportation.<br>Inconvenient and lack of time.<br>Difficulty accepting the diagnosis.<br>No direct benefit (e.g. didn't want placebo.)<br>Primary Barriers:<br>WA: Discomfort and inconvenience.<br>AA: General distrust of the research process, and reluctance to seek medical care.	Out of the focus groups emerged the following themes to inform future recruitment strategies:<br>• Inclusion of care givers in the recruitment process and research findings.<br>• Social and emotional support from research staff was 'an unexpected benefit' of the research experience for both white and AA caregivers.	No views from those who have refused research participation.	Age<br>Ethnicity
Use of live theatre to increase minority participation in Alzheimer's disease research (Fritsch *et al.* 2006) **[69]**	USA<br>African American ethnicity<br>Please see paper for specific demographic information on who attended each play.	The effects of two plays on two sets of outcomes:<br>Play One: To determine if recruitment of African American's could be enhanced in the University Memory and Aging Centre (UMAC) research registry.	Distrust	None discussed.	Play One: In the five years before the play the minority recruits were 17% compared with 36% six months after the it (χ^2 = 20.95, p < 0.001).<br>Play Two: The knowledge of Alzheimer's of audience members was better than non-audience members and there attitudes	The use of live theatre may not be a practical way for most research teams to access minority communities.<br>Mixed methods research as may	Ethnicity

Appendix 6
Table 5: Barriers to Recruitment with regards to Dementia.

**Paper (authors)**	**Country of Origin/ Study population**	**Methods/ study design**	**Anticipated barriers**	**Barriers reported on recruitment**	**Strategies to over come these barriers/results [Proposed, Tested and, Used Strategies]**	**Methodological limitations.**	**Primary factor discussed/**
		Play Two: To evaluate the educational effects of knowledge and appreciation of the value of research.			towards research were more positive.	have been useful as qualitative methods may have been able to elicit more views form the community.	
Effective Recruitment Strategies for Latino and Caucasian dementia family care givers in intervention research (Gallagher-Thompson *et al.* 2004) **[62]**	USA<br>N=121<br>Latino = 61<br>Caucasian = 60<br>Age not listed	Psychological Intervention: (RCT) Comparison of recruitment methods for RCT.<br>1) Non-professional e.g. health fairs, friends, word of mouth.<br>2) Professional community agencies, e.g. health care agencies<br>3) Media Advertisements e.g. flyers, public service announcements on radio and TV and internet notices.	Stigma regarding mental illness and help seeking, lack of information about dementia, language barriers, and transportation difficulties.	Caucasians were significantly more likely to be recruited across all strategies (χ^2 [1] = 11.28; p = 0.001).<br>Non- professional referrals and media referrals were less effective for Latinos than Caucasians (χ^2 [1] = 5.91; p = 0.015).	Bilingual and bi cultural staff employed, media advertising directed at the community.	Data on those who refused participation was not collected.	Ethnicity
Recruiting Chinese Americans for dementia caregiver intervention research: suggestions for success	USA<br>Chinese American (CA) N = 45<br>White American (WA) N = 67<br>Age not listed	Psychological Intervention study: Examination of recruitment source and participant's enrolment in study.<br>1)Media advertisements<br>2) Non-professional referral sources e.g.	Distrust of research, potential harm to the elderly, perceived lack of benefits to the family.	CA were significantly less likely to enrol in the intervention than whites (χ^2 = 4.98, p < 0.05).	Referrals from professionals yielded the highest rates of actual study enrolment for the CA participants, whereas relatively few enrolled who contacted us from non-professional sources.	No direct assessment about barriers to participation was done.	Ethnicity and Age

Appendix 6
Table 5: Barriers to Recruitment with regards to Dementia.

**Paper (authors)**	**Country of Origin/ Study population**	**Methods/ study design**	**Anticipated barriers**	**Barriers reported on recruitment**	**Strategies to over come these barriers/results [Proposed, Tested and, Used Strategies]**	**Methodological limitations.**	**Primary factor discussed/**
(Gallagher-Thompson *et al.* 2006) **[29]**		friends, word of mouth, community workshops.<br>3) Professional referrals.					
Gaining and sustaining minority participation in longitudinal research projects. (Gauthier and Clarke, 1999) **[24]**	USA<br>African-American with Alzheimer's Disease.<br>N = not listed.	A description of the Boston University Alzheimer's Disease Centre (BUADC) in relation to recruitment and retention of African-American's.	Mistrust of healthcare system.<br>Lack of awareness about trials.<br>Economic factors.<br>Fear of drug side effects.<br>Researcher's inability to understand patient's language or culture.	Outreach worker identified barriers in the community:<br>• Distrust of the medical establishment in Boston.<br>• Fear of being 'used'.<br>• Fear of being identified as having dementia or Alzheimer's disease.	BUADC Approach:<br>Recruit patients using an existing relationship with the Boston University Medical Centre Geriatric Service.<br>And build on the researcher's association with a neighbourhood centre, support given to community events<br>Outreach Worker:<br>A person who lived in the community to guide and recruit participants.	No recruitment figures therefore unable to determine how effective these strategies were.	Ethnicity.
Lessons learned from international comparative cross-cultural studies on dementia (Hendrie, 2006) **[66]**	USA/CANADA/ CHINA<br>Three minority groups:<br>Blacks from Indianapolis,<br>Cree and English-speaking residents in Manitoba, and<br>Rural Chinese.<br>Age >65<br>N = not listed.	Community survey:<br>Screening for prevalence of dementia across three minority groups	Mistrust of government related agencies and health care services.	None discussed.	Recruitment practices for Cree population discussed:<br>Employed a Cree public health nurse, living on the reserve, a native American psychologist well known to the community.<br>Culturally relevant measures developed and employed translation of the Alzheimer screening measures.<br>Met with tribal leaders to discuss project, they were updated on study progress and result.<br>Results: Refusal rates for the elderly Cree Indian for the AD		Ethnicity<br>Age

Appendix 6
Table 5: Barriers to Recruitment with regards to Dementia.

**Paper (authors)**	**Country of Origin/ Study population**	**Methods/ study design**	**Anticipated barriers**	**Barriers reported on recruitment**	**Strategies to over come these barriers/results [Proposed, Tested and, Used Strategies]**	**Methodological limitations.**	**Primary factor discussed/**
					research was 8% (15).		
Selection bias during recruitment of elderly subjects from the general population for psychiatric interviews. (Heun *et al.* 1995) **[35]**	GERMANY<br>60 – 90 years.<br>N = 291 (interviewed)<br>M = 78.9 (interviewed)	Epidemiologic investigation into the Elderly: Stratified sample of 1305 were contacted via mail and then telephone to obtain consent to participate in a psychiatric interview.	None discussed.	• People most likely to participate were younger, male, and married.<br>• Subjects who were interviewed after the first approach had fewer psychiatric disorders than those who were interviewed after a second or third approach (lifetime diagnosis of a psychiatric disorder: 22.3% vs 36.2%, $x^2 = 6.42$, $df = 1$, $P = 0.01$).<br>Barriers speculated to be related to recruitment: Information letter may not meet all of the information needs of the potential participants, description of disadvantages (2 hour commitment) didn't outweigh advantages (help science), and possibility of immediate refusal with know explanation.	Proposed Strategy:<br>Clinician referred or hospital based recruitment may yield higher recruitment rates.	Data on ethnic group was not obtained<br>Simple overview of the sample they managed to recruit rather than explicitly looking at barriers and strategies.	Age<br>Gender
Working with culture: A qualitative analysis of barriers to the recruitment of	USA<br>Chinese American family caregivers living in Boston<br>N = 25<br>Age not listed.	Qualitative Analysis based on interviews and a collection of field notes by researchers and project ethnographers drawn from a wider	None discussed.	Four main themes concerning barriers to participation:<br>• Dementia related changes construed as a normal part of aging.<br>• Research participation	No explicit strategies to address the identified barriers were discussed.	No systematic methodology on how the presenting themes were extracted from the various sources of	Ethnicity and Age.

Appendix 6
Table 5: Barriers to Recruitment with regards to Dementia.

**Paper (authors)**	**Country of Origin/ Study population**	**Methods/ study design**	**Anticipated barriers**	**Barriers reported on recruitment**	**Strategies to over come these barriers/results [Proposed, Tested and, Used Strategies]**	**Methodological limitations.**	**Primary factor discussed/**
Chinese-American family caregivers for dementia research. (Hinton *et al.* 2000) **[51]**		study on dementia care giving by the Harvard Exploratory Centre.		was viewed as potentially harmful because it can lead to excessive worry.<br>• Social stigma of Alzheimer's disease in Chinese communities.<br>• Clinician's tendency to avoid formal dementia diagnosis (and the impacting of labelling patients), instead supporting culturally congruent explanations for behaviours.		data.	
Non- response pattern bias in a community-based cross-sectional study of cognitive function among the elderly (Launer *et al.* 1994) **[36]**	HOLLAND<br>Age stratified sample (65-74, 75-84 years) drawn from patients who had refused participation in the Amsterdam Study of the Elderly (AMSTEL) recruited from 8 Medical Practices N = 115<br>Participants in AMSTEL recruited from the same 8 Medical Practices.	Patients were interviewed and sociodemographic data, health status, and mental functioning (same questions as AMSTEL) information was collected.<br>Patients who were 'non-responders' or 'responders' to were compared.	None discussed.	Non responders to the survey were significantly more likely to report a history of psychiatric illness (24.5 % v 17.7% odds ratio=1.6 95% confidence interval 1.0-2.7). They were also more likely to have had a history of stroke or diabetes and were likely to be unmarried, have lower education.<br>Non –responders aged 65-74 performed poorly on the cognitive test compared to responders: the odds of a non-responder performing poorly was 4.2 (95% CI 1.5-	None discussed.	Despite interviewing non-responders no direct questions about the reasons for their non participation were asked.<br>No data on ethnicity.	Age

Appendix 6
Table 5: Barriers to Recruitment with regards to Dementia.

**Paper (authors)**	**Country of Origin/ Study population**	**Methods/ study design**	**Anticipated barriers**	**Barriers reported on recruitment**	**Strategies to over come these barriers/results [Proposed, Tested and, Used Strategies]**	**Methodological limitations.**	**Primary factor discussed/**
	N = 999			11.5) .No difference between non-responders aged ≥75 group.			
The matching model of recruitment. (Levkoff *et al.* 2000) **[52]**	USA<br>Experiences of researchers recruiting ethnic minority care (African American, Chinese American, Latino, Irish American) givers of elders with dementia into a study.<br>N and Age not listed.	The paper presents a model of ethnic minority recruitment formulated after discussions with Exploratory Centre researchers and ethnic minority community members.<br>The model consists of two perspectives (researchers/ minority group) of which there are three levels in each (macro/institutional, mediator/gatekeeper, micro/individual). The reported barriers and strategies are described in reference to this model.	Not Applicable.	Ethnic Minority Group Perspective:<br>Macro level<br>• High service demands<br>• Employee turnover<br>Mediator level<br>• Patient's family and clinicians resist from referring in an effort to protect clients and avoid social stigma.<br>Individual level:<br>• Distrust of research and sceptical of confidentiality of interviews.<br>• Fear of stigma to the family and loss of services.<br>Researcher Perspective:<br>Macro level<br>• Academic centres competing to study the same groups<br>• Tensions between academic centres and local agencies (university	Ethnic Minority Group Perspective:<br>Macro level<br>• Want to improve community and gain something.<br>Mediator level:<br>• Believe in importance of research.<br>• Readily identify participants.<br>Individual level:<br>• Want to share stories and improve life.<br>Researcher Perspective:<br>Macro level<br>• Offer technical assistance for community generated projects.<br>• Recognise local and cultural beliefs<br>Mediator level:<br>• Interdisciplinary differences that benefit research<br>Individual level:<br>• Use of ethnography and qualitative research	The methods of analysing the interviews are not explained clearly. This makes the findings extremely difficult to extrapolate.	Ethnicity

Appendix 6
Table 5: Barriers to Recruitment with regards to Dementia.

**Paper (authors)**	**Country of Origin/ Study population**	**Methods/ study design**	**Anticipated barriers**	**Barriers reported on recruitment**	**Strategies to over come these barriers/results [Proposed, Tested and, Used Strategies]**	**Methodological limitations.**	**Primary factor discussed/**
				perceived as an elite power structure)<br>Mediator level:<br>• Interdisciplinary differences.<br>Individual level<br>• Value own activist agenda over the research agenda. (Refuse to interview people without assisting them as well).	• Adapt research<br>• Rely on existing networks<br>• Language skills<br>.		
Carer Impressions of participation in Alzheimer's disease clinical trials: What are their hopes? And is it worth it? (Mastwyk *et al.* 2002) **[59]**	AUSTRALIA<br>Carers of participants in three industry-led clinical trials for Alzheimer's Disease.<br>N = 25<br>Age not listed.	Survey to determine why carers of people with Alzheimer's Disease seek the participation of their relatives in clinical trials.	None discussed.	None discussed.	The results of the survey suggest that recruitment strategies should focus on the main motivating factors reported by carers:<br>• Improving the health of the patient.<br>• Contributing to medical science indicating that the availability of follow up after a trial is an important incentive.		Age
Characteristics of non-responders and the impact of non-response on estimates of	USA<br>Utah heads of households > 75.<br>People who agreed to participate after	Initial Responders and Initial Non-responders in the cross sectional community survey were compared.	None discussed.	Initial responders had higher educational attainment than initial non-responders.<br>Initial non-responders reported lower cognitive	Researchers suggest the use of face-to-face contact with subjects and sensitive interviewers who are capable of explaining the study clearly and allaying any fears.	The characteristics of the initial non-responders are unable to be generalised to	Age

Appendix 6
Table 5: Barriers to Recruitment with regards to Dementia.

**Paper (authors)**	**Country of Origin/ Study population**	**Methods/ study design**	**Anticipated barriers**	**Barriers reported on recruitment**	**Strategies to over come these barriers/results [Proposed, Tested and, Used Strategies]**	**Methodological limitations.**	**Primary factor discussed/**
dementia (Norton *et al.* 1994) **[60]**	being contacted by letter and/or phone (initial responders) N = 82.<br>People who agreed to a participate only after being followed up by a home visit (initial non-responders) N = 37			function on the Mini-Mental State Examination.		other people who refuse research participation.<br>Non representative sample, mainly white (99.1%) and male (82.4%).	
Recruiting patients for drug trials: A difficult task (Skerritt *et al.* 1996) **[63]**	ENGLAND<br>Dementia patients: Mini Mental State Examination score between 10 and 26.<br>N= 41	Drug trial	Physical illness, Infirmity, Isolation.	Low recruitment rates:<br>150 letters sent to GP: 2 Recruits.<br>Out-patients department at Hammersmith hospital: 4 recruits.<br>Advert in Alzheimer's Disease Society Newsletter: 2 recruits.<br>Memory Clinic Hammersmith Hospital: 31 Recruits.	No explicit strategies based on these low recruitment rates are suggested other than a focus on places like the memory clinic where more motivated patients are likely to be identified.	No information on ethnicity.	Age
The Medicare Alzheimer's project, in Portland Oregon (Warren *et al.* 1991) **[40]**	USA<br>Article discusses the recruitment of Alzheimer's Disease patients and their families into a large federal research project. Aim to recruit 600	Treatment group received case management, day care, and respite care, home health care, meals, medical and incontinence supply's, chore service, durable medical equipment,	No major barriers were anticipated.	• No financial reward offered to control groups.<br>• Project too lengthy<br>• Death or placement in long term care facility<br>• Study forms were described as	Following insufficient response, an outreach worker was hired; focusing on contact with a physician who would send potential patients letters about the study, stating that a researcher would be calling them. This effort increased recruitment significantly as patients had to actively opt out		Age

Appendix 6
Table 5: Barriers to Recruitment with regards to Dementia.

**Paper (authors)**	**Country of Origin/ Study population**	**Methods/ study design**	**Anticipated barriers**	**Barriers reported on recruitment**	**Strategies to over come these barriers/results [Proposed, Tested and, Used Strategies]**	**Methodological limitations.**	**Primary factor discussed/**
	in Oregon, over 3 years with Alzheimer's Disease and reversible dementia. > 65. Eight recruitment sites were selected this paper discusses the programme at good Samaritans hospital and medical centre in Portland, Oregon	transportation, family education and training at no cost to the families. Control group receive no project services, but evaluators interviewed them every six months.<br><br>Paper does not describe the duration of the projects.		overwhelming to the care giver.<br>• Husbands with dementia often refused, even if a wife caregiver wanted to participate.<br>• Families did not want to expose loved ones to tests	of being contacted.<br><br>The term Alzheimer's Disease may reduce recruitment, terms 'memory loss' and 'confusion' may have resulted in increased recruitment.		
